# BIOZON: a system for unification, management and analysis of heterogeneous biological data

**DOI:** 10.1186/1471-2105-7-70

**Published:** 2006-02-15

**Authors:** Aaron Birkland, Golan Yona

**Affiliations:** 1 Department of Computer Science, Cornell University, lthaca, NY, USA

## Abstract

**Background:**

Integration of heterogeneous data types is a challenging problem, especially in biology, where the number of databases and data types increase rapidly. Amongst the problems that one has to face are integrity, consistency, redundancy, connectivity, expressiveness and updatability.

**Description:**

Here we present a system (Biozon) that addresses these problems, and offers biologists a new knowledge resource to navigate through and explore. Biozon unifies multiple biological databases consisting of a variety of data types (such as DNA sequences, proteins, interactions and cellular pathways). It is fundamentally different from previous efforts as it uses a single extensive and tightly connected graph schema wrapped with hierarchical ontology of documents and relations. Beyond warehousing existing data, Biozon computes and stores novel derived data, such as similarity relationships and functional predictions. The integration of similarity data allows propagation of knowledge through inference and fuzzy searches. Sophisticated methods of query that span multiple data types were implemented and first-of-a-kind biological ranking systems were explored and integrated.

**Conclusion:**

The Biozon system is an extensive knowledge resource of heterogeneous biological data. Currently, it holds more than 100 million biological documents and 6.5 billion relations between them. The database is accessible through an advanced web interface that supports complex queries, "fuzzy" searches, data materialization and more, online at .

## Background

High throughput technologies such as microarrays and mass spectrometry, as well as fast sequencing techniques produce biological data at an ever increasing rate. The sheer volume of new data exposes new processes and complex phenomena in biological systems. Consequently, the focus is shifting from exploring single molecules to complexes of molecules or pathways involving multiple proteins and other subcellular agents. Often, the study of one entity is tightly coupled to the study of other, related entities. For example, by studying individual proteins we wish to better understand their role in cellular processes, and by studying cellular processes, we hope to better understand cellular "computations", and to gain insight into the functions of the constituent molecules.

With the constant flow of new data, biological data analysis becomes a major challenge. The massive amount of available data rules out comprehensive experimental research of all known proteins, pathways and other biological entities. In this view, advanced and automatic tools to organize and analyze this data become a necessity. Acknowledging this need more than two decades ago, efforts have been made at collecting and storing biological data on digital media, and today there are many databases that warehouse a variety of biological knowledge. Some are collections of fundamental biological entities and annotations, such as the protein sequence databases SwissProt [1] and PIR [2] or the Genbank database of DNA and RNA sequences [3]. Databases of protein interactions such as BIND [4] or DIP [5] provide insight into the basic processes of the cell, while sources such as the metabolic pathway databases EcoCyc [6] and KEGG [7] describe *systems *that are comprised of these basic processes, and other cellular agents. This is a just a short list of the many biological databases that are available today (see NAR database issue at [8]). Existing databases are typically highly focused, containing raw data of a specific type and annotations from recent scientific research and publications. However, entities that are stored in different databases can be strongly related and mutually dependent on each other, and to fully understand the role of an individual entity one has to know *which *and *how *other entities are related to it. For example, the function of genes depends on their broader biological context: their relations to other genes, the set of interactions they form, the pathways they are involved in, their expression under various conditions, and so on. In a similar manner, the biological function of an interaction is a function of the interacting partners. Utilizing this interrelated information is key to an effective and accurate analysis of biological entities.

To retrieve the broader context of an entity, a biologist usually has to search multiple databases, facing several obstacles. Most of the data in these databases is publicly available as semi-structured text files or custom web interfaces, and to obtain the relevant data one has to query each database independently (online or by downloading and parsing text files) and then unify this knowledge into a consistent and non-redundant set. This task is time-consuming and can be surprisingly difficult. Existing databases use explicit references by accession number or a mutual ontology to identify entities, and each database uses its own set of identifiers. Some databases relate and cross link elements from other databases based on these identifiers, but this information is very partial. Moreover, these links are not always established in coordination between databases. As biological databases change rapidly, this inevitably creates problems of consistency, synchronization and updatability. Therefore, even if possible manually on a small scale (for a given protein or interaction, for example), data integration becomes a daunting task for anything that involves more than a few individual entities.

The problem of data integration is most pronounced when querying data. Given the distributed nature of the source data, and the lack of structured mechanisms for forming cross-links between them, it is difficult to mine biological data while leveraging from the mutual dependency between entities. To overcome this limitation it is necessary to create an infrastructure for a unified biological knowledge resource that would seamlessly integrate data from different resources and aspects of biological systems. A gold standard of biological data integration should allow one to see an instance of such data in its full biological context. More importantly it would allow for complex searches that span multiple data types from a heterogeneous set of sources and allow for arbitrary computations on that data.

Integration of biological databases has been an ongoing research problem. There are several approaches and degrees of freedom in designing a practical system, as detailed in [9,10], including the degree of federation and the choice of warehoused, instantiated data vs. views on distributed, independent sources. Current methods differ greatly in their aims and scope. As was noted in [11], solutions in this area can be generally classified into three main categories: portal, mediator, and warehouse. *Portal *oriented systems are mainly navigational. These systems perform fast, indexed keyword searches over a flat set but do not actually integrate the data itself and relationships between data items in these tend to be link driven. Examples of such systems are SRS [12] and Entrez [13]. *Mediator *oriented systems use a mediated schema and/or wrappers to distribute queries amongst different sources, integrating the information in a middle layer. Examples are DiscoveryLink [14], BioMediator [15], TAMBIS [16], OPM [17] and others [18-22].

These systems provide a qualified mediated schema onto which sources are mapped, or a single interface or language for access and operations on data from heterogeneous sources, such as CPL [23] or sSQL. However, efficiency, speed, and data availability are major issues with all mediated solutions. This is a substantial drawback when such performance criteria are significant. Large joins in particular are almost always guaranteed to be slow in non-warehoused environments, and unfortunately these are usually important when executing complex queries over large result sets. *Warehouse *oriented systems integrate data into a locally warehoused environment. This is the category that Biozon belongs to, and it includes a few other efforts such as GUS [18] and its derivatives [24]. Warehouse systems enable more control over query optimization and execution, and allow data manipulation and exploration to an extent that is not possible with other approaches (a detailed discussion appears in the the 'Related Studies' section of the Supplementary Material).

While a great first step to increasing the utility of the available data, currently existing methods are not entirely complete. To the best of our knowledge, no current solution implements integration to its full extent such that the overlapping nature of the data is addressed. Indeed, most existing solutions achieve "horizontal integration", which treats data sources as mostly complimentary, and ignores issues that are associated with data aggregation [11]. Our challenge was to develop a database infrastructure that addresses all the aforementioned issues and that considers the overlapping nature of data such as to expose the broader biological context of entities. Furthermore, our goal was to design a system that enables methods of complex query and navigation, including realtime execution of "fuzzy" queries that rely on similarity relations and ranking engines that exploit high-order structure in the data.

In the next sections we lay out the main elements of Biozon. We start with a description of the data model and design choices, and follow with our specific integration methodology. With these principles established, we continue on to discuss current applications such as complex and fuzzy queries on the graph data, graph topologies, and analysis of graph edges that allows for ranking of search results. Lastly, we conclude with a brief mention of our implementation, discussion, and identify areas for future research or added capability. A description of the user interface and the main database features appears in [25].

### Construction and content

There are at least two degrees of freedom when designing a system for data integration: Where and how the data is accessed (a view over external federated sources vs locally stored, instantiated data), and the specificity of the overall schema (loose vs. tight highly-structured schema). The combination of a tightly integrated schema with locally instantiated data produces the greatest benefits [9], although at the added cost of storage and maintenance. Practically, complex searches and large scale computations on live and changing data are only feasible with a tightly integrated schema where data from all sources are present in a single location. Since our goal was to be able to store the results of expensive computations on source data as well as allowing advanced search and navigation across data types, we were compelled to adopt such a scheme.

There are several main elements that guide our design. We seek a data model that (1) tightly integrates multiple data types (2) that can be easily expanded to represent new data types (3) that is consistent with the source databases, and (4) that is highly expressive, allowing complex searches and data propagation. Additionally, the model needs to be simple enough such that it can be relatively easily implemented and extended, and shared by the scientific community.

### Data model

There are two common approaches toward representing heterogeneous biological data. The first relies on hierarchical models [21,26] while the other on graph models [6,7]. Hierarchical models have the advantage that entities can inherit properties from the parent types, thus simplifying maintenance and expandability. These structures are also more amenable for certain types of searches and are conceptually easier to comprehend because of the way knowledge is delineated, classified and ordered. However, hierarchical representation cannot fully encompass the complexity observed in biological systems. A simple example is the gene ontology database [26] that was created by experts, in an attempt to standardize the nomenclature used for functional annotations of biological entities. Despite the overall hierarchical structure, many entries in this classification deviate from the traditional tree hierarchy, and posses multiple parents, thus mitigating some of the advantages associated with strictly hierarchical models. Graph models, on the other hand, are more general and can describe complex structures with different types of dependencies other than just child-parent. Interrelated life sciences data is especially well suited to being represented as a graph, as is evidenced by projects such as KEGG [7] or MetaCyc [6].

In this view, we chose to employ a synergistic approach and base the Biozon infrastructure on a combination of a more expressive core graph model supported with a class hierarchy imposed on each graph element. These two components serve to characterize different aspects of our system (global structure of interrelated data vs. the nature of individual data entities). The combined approach provides a flexible solution that can be adjusted in multiple ways to best describe arbitrary biological entities. Each biological entity can be expressed and characterized by either introducing more constraints on its nature in the hierarchy or on the structure of its relations to other entities on the graph.

### Graph model: logical structure and schema design

The data in Biozon is represented as a graph in a design that has parallels to an entity-relationship model. In the **data graph **∑, each node represents some entity (e.g. a protein sequence, a pathway or a descriptor document) and an edge between two nodes represents a relationship between them. We use the term **document **to refer to a graph node, and the term **relation **to refer to a graph edge. Formally, ∑ = (**V**, **E**) where **V **= {*v*_1_, *v*_2_...} is the set of all nodes (documents) in the graph, and **E **= {*e*_1_, *e*_2_...} is the set of all edges **E **⊂ **V **× **V**.

**Figure 1:**
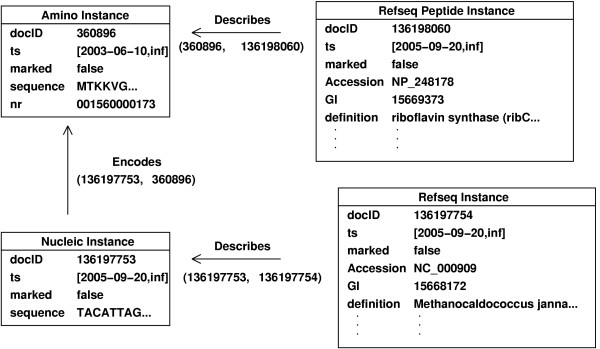
**Document Instances**. Abbreviated instances of an amino acid and nucleic acid sequence objects with their respective descriptors, as mapped to the Biozon data graph from a single RefSeq document. The two objects are related by an 'encodes' relation *e *= (136197753, 360896), and are each related to descriptor annotation separately through 'describes' relations.

Documents as graph nodes are a fundamental unit of data in the Biozon database. Every document contains set of attribute-value pairs that represent some unit of biological data, and each document is assigned a globally unique identifier called a docID. Relations are the edges that connect the documents of the data graph, and likewise may have attributes that define and refine the nature of the relation. Relations in Biozon are directional, consisting of a **referring document and a referred document**. A relation *e *between referring document *v*_1 _and referred document *v*_2 _can thus be represented as an ordered pair *e *= (*v*_1_, *v*_2_). Figure 1 shows an example of document instances connected by a relation on the graph. Data from any given source is represented in Biozon as a set of nodes and edges on the Biozon graph. Figure 1 for example, indicates how a single RefSeq [27] document is instantiated on the Biozon graph as four interrelated documents.

While simple and straightforward, the graph representation is amenable to special operators and efficient graph algorithms that can be used in data integration, mapping, propagation, and updates, as is discussed in the next sections. Moreover, this structure can be easily expanded and it is conducive to searches and queries that span multiple data types that are related together through graph edges.

### Hierarchical classification and semantics

The hierarchical element of our model is implemented as a partial order over documents (nodes) and relations (edges) that serve to organize domains of knowledge into classes and subclasses, to aid in the development of new classes and to simplify maintenance protocols.

### Document types and document classification

**Figure 2:**
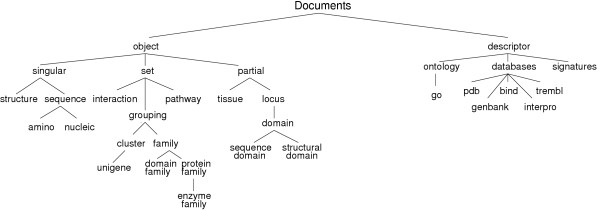
**A partial snapshot of the Biozon hierarchical document classification model**. A major distinction is made between descriptors and objects (see text for details). The presence of a particular class in the hierarchy can arise due to physical or semantic differences in the nature of the documents therein. For example, amino acids and nucleic acids are both stored as text strings in the database and their internal representations are identical (although over different alphabets). However, they represent fundamentally different real-world objects and should be classified as such. A special subclass of objects is **locus**. This type serves to localize information with respect to larger objects or to represent efficiently objects that are essentially sub-entities of other existing objects (for example, a protein domain is a locus with respect to a protein sequence, with specific start and end positions).

There are different types of documents in Biozon, as listed in Table 1 To define the biological context of documents we construct a document classification hierarchy that corresponds to different domains of knowledge. Every class of documents represents a distinct data type or a generalized data type, and every document is classified at some level of this hierarchy (Fig. 2) based on its meaning, content or origin. Every class in the hierarchy tree relates to its parent through an 'is a' relationship, and inherits the properties of its ancestor class (for example, its set of attributes). The inheritance also allows sharing functional elements with ancestor document types. One such example is object comparison operators, as discussed in the 'Data Integration' section.

**Table 1:** Document Types in Biozon. Each type is represented differently in Biozon's implementation. Each representation may be decomposed into a number of atomic units for the purpose of comparison.

Document type	Representation	Atomic units
protein sequence	string	amino acids
nucleic acid sequence	string	nucleic acids
protein family	set	proteins
pathway	set	protein families
domain	ordered pair	sequence coordinates
domain family	set	domains
interaction	set	proteins, nucleic acids
descriptor	text	characters
structure	list	3D coordinates
unigene cluster	set	nucleic acids (ESTs)

The set of classes are determined by structural or semantic differences between the data represented in each graph part. We define the root node of the document class hierarchy to be simply a 'Document'. The root Document class has three attributes which are inherited by all other subclasses, namely 'docID', 'timeline' and 'marked'. The docID is a globally unique identifier, while timeline is an attribute that indicates the relevant time frame of the document. This timeline defines the visible time context of an object on the Biozon graph. Whenever an object is added to the graph, a timeline is created that starts the moment it was inserted, and extends to infinity. When it is deleted, the timeline is ended. This allows viewing snapshots of the database at any specified time. (The time context only demarcates to a document's existence in Biozon, and it is not related at all to a dataset's publication date or version number. For more information, please refer to the Supplementary Material.) The third attribute ('marked') is used as part of the maintenance protocols when deleting documents, and is discussed in the Supplementary Material (see section 'The primitive functions: maintaining internal consistency').

The first and perhaps most fundamental class difference between documents as visible in Fig. 2 is between objects and descriptors. **Objects **are documents that define a physical entity (e.g. an amino acid sequence), a logical entity (e.g domain), or a set thereof (e.g. a protein family). They contain the minimal set of attributes that is sufficient to define their physical properties and distinguish them from other objects of the same data type. This has important consequences in data integration and updates as discussed in the 'Data Integration' section. **Descriptors**, on the other hand, contain facts, conjecture, measurements, or other information that serves to describe some object in Biozon. The data in descriptors originates from annotation as well as raw measurements such as expression data that is associated with an mRNA sequence. Additional levels in this hierarchy refine classes based on physical or semantic differences. Beyond biological context, this classification serves additional purpose, as it can aid in semantic schema mapping and integration. It provides a guideline for expanding or refining the set of possible data types when integrating new data into Biozon. By traversing the tree, starting from the root node, one can map an external data type to the closest semantically related class and extend the hierarchy accordingly, if needed.

### Relation types and classification

**Figure 3:**
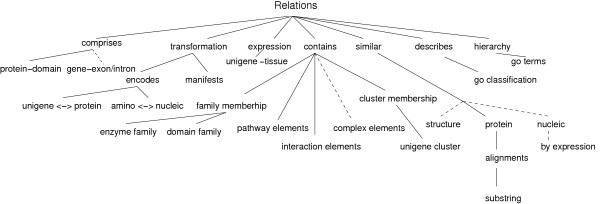
**A partial snapshot of the Biozon hierarchical relation classification model**. The primary motivation for the partitioning of the hierarchy is a difference in the semantic meaning of relationships between documents. Expansion of this hierarchy is expected as new relationships are added. Planned additions in the near future are shown as dashed lines.

Knowing *how *two documents are related is just as important as knowing that they *are *related. There are different types of relations in the Biozon database, listed in Table 2. To organize this knowledge in a modular way, we create a classification hierarchy on the relations in much the same way that we did for documents (Fig. 3), such that the semantics of each relation is determined by its class. In a similar fashion, we define the root node for this hierarchy to be 'Relation', indicating only the most general fact that two documents are related. This class is associated with the attributes 'referring', 'referred', 'timeline' and 'authority'. The authority attribute is important in updates and is discussed in the Supplementary Material (section: 'The primitive functions: maintaining internal consistency'). As with the document hierarchy, subclasses of relations inherit the properties of their ancestors. Therefore, these four basic attributes are inherited by all relation subclasses.

**Table 2:** Relation types in Biozon.

Relation type	Referring document	Referred document
manifests	protein	structure
describes	descriptor	any object
encodes.nucleic	nucleic acid	protein
encodes.unigene	unigene cluster	protein
similarity	protein	protein
contains.unigene	unigene cluster	nucleic acid
contains.interaction	interaction	protein, DNA
contains.pathway	pathway	enzyme family
contains.enzyme-family	enzyme family	protein
contains.domain-family	domain family	domain
comprises.domains	domain	protein
expresses.unigene	unigene cluster	tissue
hierarchy.go	go term	go term
describes.go	go term	protein

The main subclasses are logically defined based on the type of the objects they relate. Relations of type 'transformation' (encodes, manifests) relate two different types of objects such that one is obtained from the other. Relations of type 'contains' relate an object that is a set to its member objects. 'Similar' relates objects of the same type, while 'describes' relates descriptors to the objects they describe. It should be noted that each relation holds true in both directions although with different semantics. For example, 'describes' relates a referring descriptor and a referred object together and implies that the descriptor describes the object. Read in the other direction, an object is described by a descriptor.

As mentioned earlier, relations may have attributes that refine the nature of the relation. For example, suppose a protein is described by a Gene Ontology term. Most gene ontology mappings are associated with some evidence code. In Biozon, this information would be contained as a field of a relation 'describes.go' is a subclass under the 'describes' relation. Likewise, relations are associated with attributes that specify the significance and extent of the similarity with respect to the related objects.

It is important to note at this point that the relation hierarchy, the document hierarchy and the graph model are all subject to certain perceptions on how biological data should be organized. Unfortunately, there is no single model that is generally accepted by all. Our design was motivated by the goals that were laid out in the Background section and has many practical benefits as exemplified throughput this paper.

The use of three model elements result in a flexible design that in practice has already proved capable of incorporating new data types.

### Non-redundant object-centric model

Objects are the backbone of our data graph. Our database objects are direct analogs to *physical entities *and *sets of entities*, since these are the essence of any biological system. Indeed, any object that can be defined by its physical properties is represented as such in Biozon and comprise a non-redundant set based upon these physical 'keys'. As an example, consider proteins and their representation in Biozon. In this case, the relevant physical property that distinguishes proteins is chosen to be the sequence of amino acids. From a computational standpoint, this is a natural choice (as sequences are the basis for many sorts of analysis, such as sequence comparison, motif search and domain analysis) and an effective way to process large and highly overlapping datasets.

In the same spirit, protein families are entities that are comprised of multiple physical objects. Pathways can be composed of both sets (protein families) and individual physical entities (specific proteins) and therefore comply with our definition of an object as well.

Central to our approach to data unification is the requirement that all objects in Biozon are non-redundant on their physical keys. We define the function *object*(*v*) that returns the key that is associated with an object document *v*. As an example, in the case of proteins, *object*() will return a sequence string that may be compared with others to determine equality. The return value can also be a set of document IDs, for example when *v *is an interaction, pathway or a protein family object that are defined uniquely based on their constituents.

The reliance on physical entities and sets of physical entities as our backbone is especially useful for data integration since it allows unambiguous unification of many entities from different databases based on their physical properties, as is discussed in the 'Date Integration' section. Such integration also results in a more comprehensive knowledge resource, since characteristics that have been identified for a certain object usually pertain to other objects (e.g. in other species) of identical physical properties. Nevertheless, despite all the advantages that this approach yields, it should be noted that from other viewpoints, entities of identical physical properties are not considered the same object. For example, even 100% identical protein sequences in different species might have different properties. This can be easily resolved by projecting the data graph onto the organism of choice (see Future Work in the Conclusion) and future versions of Biozon will allow one to view an entity in its organism-specific context, derived through such projections.

### Source and derived data

**Figure 4:**
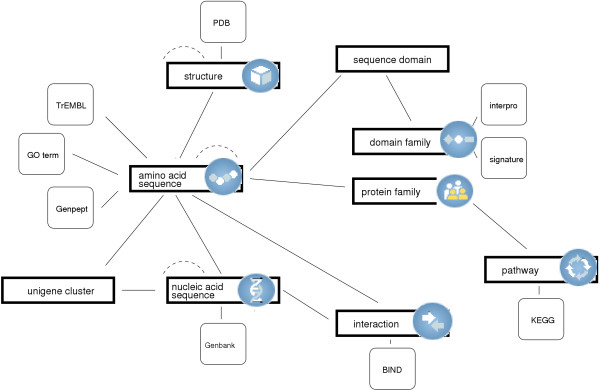
**Partial overview of the Biozon schema**. Similarity relations are depicted with dashed lines. The database will be gradually extended to span both new source data types as well as new derived data.

A partial overview of the current schema is given in Fig. 4. Much of the data in Biozon is gleaned from publicly available databases such as SwissProt PDB, Genbank, BIND, KEGG, and more. We refer to this type of data as **source data**. These sources provide the fundamental biological objects in Biozon, many of the relationships that exist between objects, and the annotation that makes it possible for humans to understand them. To avoid issues of intellectual property we host only the data that is publicly available, with proper credits and copyright endorsements. There are some databases that have restrictions on the use and distribution of their data, such as DIP and HPRD. Data from these sources may be incorporated into Biozon for research purposes in-house, but are not allowed to be viewed by the public. Biozon is able to represent a minimal skeleton of protected (private) data with links to the full details on its originating source in cases where such a scheme is appropriate. To protect private data Biozon uses a user account system where each user belongs to one or more user classes. Each class is mapped to a different subspace in Biozon and enables its members to access documents only in that subspace.

**Derived data **encompasses any data that is produced as the result of a computation or operation over some set of existing data in Biozon, and is unavailable elsewhere. Currently, derived data available in Biozon consists of similarity relations between protein sequences and protein structures, domain structure of proteins and more (see 'Derived Data' section). A specific type of derived data is *derived relation*. For example, classifying a protein to an Enzyme family is often based on analysis of the descriptors associated with that protein. Such an analysis creates a relation ('contains.enzyme-family') between the enzyme family and the protein. In general, a derived relation is of the form *e*_*d *_= *f*(σ), where *f *is some function that accepts part of the graph σ ⊂ ∑ as an input and returns a relation as output. Derived relations play an important role in data integration in Biozon because they provide a concrete and materialized method of indicating relationships that are otherwise not necessarily obvious.

As of October 2005 Biozon contains more than 42 million DNA sequences (from Genbank and RefSeq), 2.8 million protein sequences (from Swiss-Prot/TrEMBL, Genpept, RefSeq, PDB, PIR, BIND), 123,000 protein-protein interactions (from BIND, PIR as well as predicted interactions) and other entities. There are about 60 million descriptors and more than 1.7 billion words indexed. Biozon also contains more than 6.5 billion derived relations based on sequence, structure and expression similarity. The complete list of source and derived data and their origin is available at [25].

### Data integration

Biozon employs a vertical integration approach, such that sources are not only incorporated into a single schema but are also integrated using a non-redundant object-centric model. The implication of this approach is that data integration entails two major steps of schema mapping and semantic data mapping. The first converts a source into a large graph over the Biozon schema. The second serves to map overlapping entities (nodes) into a non-redundant set. This design choice has many benefits as is laid out in this and the next sections.

### Definitions

The schema level description of Biozon is a skeleton graph as depicted in Fig. 4. Formally, *schema*_∑ _= (**C**_*V*_, **C**_*E*_), where **C***v *= {c1v,c2v
 MathType@MTEF@5@5@+=feaafiart1ev1aaatCvAUfKttLearuWrP9MDH5MBPbIqV92AaeXatLxBI9gBaebbnrfifHhDYfgasaacH8akY=wiFfYdH8Gipec8Eeeu0xXdbba9frFj0=OqFfea0dXdd9vqai=hGuQ8kuc9pgc9s8qqaq=dirpe0xb9q8qiLsFr0=vr0=vr0dc8meaabaqaciaacaGaaeqabaqabeGadaaakeaacqWGJbWydaqhaaWcbaGaeGymaedabaGaemODayhaaOGaeiilaWIaem4yam2aa0baaSqaaiabikdaYaqaaiabdAha2baaaaa@355A@ ...} is the set of document classes (for example, **C***v *= {structures, interactions, protein sequences, SwissProt documents, ...} as described in the section on 'Document Types and Classification') and **C**_*E *_= {c1e,c2e
 MathType@MTEF@5@5@+=feaafiart1ev1aaatCvAUfKttLearuWrP9MDH5MBPbIqV92AaeXatLxBI9gBaebbnrfifHhDYfgasaacH8akY=wiFfYdH8Gipec8Eeeu0xXdbba9frFj0=OqFfea0dXdd9vqai=hGuQ8kuc9pgc9s8qqaq=dirpe0xb9q8qiLsFr0=vr0=vr0dc8meaabaqaciaacaGaaeqabaqabeGadaaakeaacqWGJbWydaqhaaWcbaGaeGymaedabaGaemyzaugaaOGaeiilaWIaem4yam2aa0baaSqaaiabikdaYaqaaiabdwgaLbaaaaa@3516@ ...} is a set of relation classes, such that each relation class *c*^*e *^∈ **C**_*E *_relates two document classes *c*^*e *^= (civ,cjv
 MathType@MTEF@5@5@+=feaafiart1ev1aaatCvAUfKttLearuWrP9MDH5MBPbIqV92AaeXatLxBI9gBaebbnrfifHhDYfgasaacH8akY=wiFfYdH8Gipec8Eeeu0xXdbba9frFj0=OqFfea0dXdd9vqai=hGuQ8kuc9pgc9s8qqaq=dirpe0xb9q8qiLsFr0=vr0=vr0dc8meaabaqaciaacaGaaeqabaqabeGadaaakeaacqWGJbWydaqhaaWcbaGaemyAaKgabaGaemODayhaaOGaeiilaWIaem4yam2aa0baaSqaaiabdQgaQbqaaiabdAha2baaaaa@3630@) where civ,cjv
 MathType@MTEF@5@5@+=feaafiart1ev1aaatCvAUfKttLearuWrP9MDH5MBPbIqV92AaeXatLxBI9gBaebbnrfifHhDYfgasaacH8akY=wiFfYdH8Gipec8Eeeu0xXdbba9frFj0=OqFfea0dXdd9vqai=hGuQ8kuc9pgc9s8qqaq=dirpe0xb9q8qiLsFr0=vr0=vr0dc8meaabaqaciaacaGaaeqabaqabeGadaaakeaacqWGJbWydaqhaaWcbaGaemyAaKgabaGaemODayhaaOGaeiilaWIaem4yam2aa0baaSqaaiabdQgaQbqaaiabdAha2baaaaa@3630@ ∈ **C**_*V*_. We denote the subset of *object *data types by **C***o *(**C***o *⊂ **C**_*V*_).

The Biozon data graph is the instance level graph ∑ = (**V**, **E**) over *schema*_∑_. We denote the class of a specific instance of a document *v *∈ **V **or a relation *e *∈ **E **by *class*(*v*) and *class*(*e*) respectively. Each document must be classified to one of the classes **C**_*V*_, i.e. ∀*v *∈ **V**, *class*(*v*) ∈ **C**_*V*_. Similarly, ∀*e *∈ **E**, *class*(*e*) ∈ **C**_*E*_.

### Mapping

Each of the sources is referred to as a database *D *that is comprised of a set of fundamental units of data referred to as records or elements *d*, such that *D *= {*d*_1_, *d*_2_, ... }. Each database might use a different data model, and in order to be integrated into Biozon, *D *must be mapped to some representation in our data graph ∑. This is a challenging problem, especially so with the diverse data types that are observed in biology, and to that end there are no algorithms that can *automatically *match an arbitrary schema over an arbitrary data model to another schema over a different data model [28].

We do not employ a single mapping language to represent the mapping specification, but instead define a generic data mapper/loader coupled with specific data transformation wrappers for each incorporated source. We first analyze the schema and semantic meaning of *D *and transform its data model to create an equivalent graph schema *schema*_*D *_that is composed of elements of **C**_*v *_and **C**_*E *_and thus can be represented in our data model. If *D *cannot be mapped using existing classes of Biozon then the relevant classes have to be added to *schema*_∑ _first.

This step of semantic schema matching is a one-time process that requires decision making in order to resolve structural conflicts (as discussed in the 'Consistency' section of the Supplementary Material). For any given *D*, there may exist several seemingly valid ways for it to be represented in S. However, it is generally desirable to transform *D *into a form that overlaps with existing document types in Biozon as much as possible. This is important from the perspective of data richness, as it will result in a graph that is most tightly interconnected due to such overlaps. For example, consider interactions. In Biozon, an interaction is represented as a set of objects that interact. There is one node on the graph representing this set, and all proteins or nucleic acids that are involved in the interaction have a relation connecting them to the interaction object. In the design process, several alternative representations were considered. For example, one alternative would be to represent interactions as 'interacts' relation between proteins. The primary disadvantages of this representation is that it would be impossible to represent interactions that involve more than two interactors (e.g. complexes). Another choice may be to keep the knowledge of interactions in some descriptors of proteins. However, that representation would not exploit the primary advantage of the Biozon model in encoding biological context in highly connected graph structure. Once *schema*_*D *_= (CV'
 MathType@MTEF@5@5@+=feaafiart1ev1aaatCvAUfKttLearuWrP9MDH5MBPbIqV92AaeXatLxBI9gBaebbnrfifHhDYfgasaacH8akY=wiFfYdH8Gipec8Eeeu0xXdbba9frFj0=OqFfea0dXdd9vqai=hGuQ8kuc9pgc9s8qqaq=dirpe0xb9q8qiLsFr0=vr0=vr0dc8meaabaqaciaacaGaaeqabaqabeGadaaakeaacqWGdbWqdaqhaaWcbaGaemOvayfabaacbiGae83jaCcaaaaa@2FFB@, CE'
 MathType@MTEF@5@5@+=feaafiart1ev1aaatCvAUfKttLearuWrP9MDH5MBPbIqV92AaeXatLxBI9gBaebbnrfifHhDYfgasaacH8akY=wiFfYdH8Gipec8Eeeu0xXdbba9frFj0=OqFfea0dXdd9vqai=hGuQ8kuc9pgc9s8qqaq=dirpe0xb9q8qiLsFr0=vr0=vr0dc8meaabaqaciaacaGaaeqabaqabeGadaaakeaacqWGdbWqdaqhaaWcbaGaemyraueabaacbaGae83jaCcaaaaa@2FD7@) has been determined (where CV'
 MathType@MTEF@5@5@+=feaafiart1ev1aaatCvAUfKttLearuWrP9MDH5MBPbIqV92AaeXatLxBI9gBaebbnrfifHhDYfgasaacH8akY=wiFfYdH8Gipec8Eeeu0xXdbba9frFj0=OqFfea0dXdd9vqai=hGuQ8kuc9pgc9s8qqaq=dirpe0xb9q8qiLsFr0=vr0=vr0dc8meaabaqaciaacaGaaeqabaqabeGadaaakeaacqWGdbWqdaqhaaWcbaGaemOvayfabaacbiGae83jaCcaaaaa@2FFB@ ⊂ **C**_*v *_and CE'
 MathType@MTEF@5@5@+=feaafiart1ev1aaatCvAUfKttLearuWrP9MDH5MBPbIqV92AaeXatLxBI9gBaebbnrfifHhDYfgasaacH8akY=wiFfYdH8Gipec8Eeeu0xXdbba9frFj0=OqFfea0dXdd9vqai=hGuQ8kuc9pgc9s8qqaq=dirpe0xb9q8qiLsFr0=vr0=vr0dc8meaabaqaciaacaGaaeqabaqabeGadaaakeaacqWGdbWqdaqhaaWcbaGaemyraueabaacbaGae83jaCcaaaaa@2FD7@ ⊂ **C**_*E*_), we use these findings to construct a transformation function *T*_*D *_that transforms the data *instances *from *D *onto ∑. For example, a *specific *RefSeq entry *d *that is represented originally as a flat record with several attributes is transformed into a small graph (as exemplified in Fig. 1) with four nodes and three edges σ(*d*) = ({*v*_1_, *v*_2_, *v*_3_, *v*_4_}, {*e*_1↔2_, *e*_2↔3_, *e*_3↔4_}), where *v*_1 _is an amino acid object, *v*_2 _is a RefSeq peptide descriptor document that contains the attributes specific to the protein sequence, *v*_3 _is nucleic acid sequence object, and *v*_4 _is a RefSeq descriptor document that contains all other attributes. Edges *e*_1↔2 _and *e*_3↔4 _are 'describes' relations, and *e*_2↔3 _is an 'encodes' relation.

Every element *d *in *D *has an analogue σ(*d*) ⊂ ∑. We define *D*_∑ _as the projection of *D *onto ∑, i.e. *D*_∑ _is the set of all subgraphs

D∑=∪d∈Dσ(d)
 MathType@MTEF@5@5@+=feaafiart1ev1aaatCvAUfKttLearuWrP9MDH5MBPbIqV92AaeXatLxBI9gBaebbnrfifHhDYfgasaacH8akY=wiFfYdH8Gipec8Eeeu0xXdbba9frFj0=OqFfea0dXdd9vqai=hGuQ8kuc9pgc9s8qqaq=dirpe0xb9q8qiLsFr0=vr0=vr0dc8meaabaqaciaacaGaaeqabaqabeGadaaakeaacqWGebardaWgaaWcbaGaeyyeIuoabeaakiabg2da9maatafabaacciGae83WdmNaeiikaGIaemizaqMaeiykaKcaleaacqWGKbazcqGHiiIZcqWGebaraeqaniablQIivbaaaa@3B04@

and ∑ is the **union **of all subgraphs from all databases *D*

**Figure 5:**
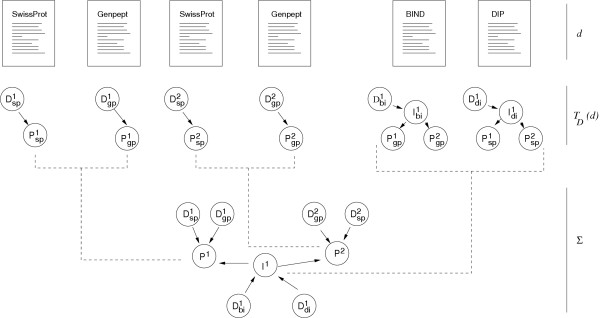
**Data integration**. Individual elements *d *from source databases are translated to their representation in Biozon as per the transformation function *T*_*D*_. The graph ∑ resulting from integration of these elements has non-redundant objects, serving to merge the data from disparate sources into a cohesive whole. As shown, six records from GenPept, SwissProt BIND and DIP are translated into Biozon graph form. Each record is transformed into a set of objects (e.g. Pgp1
 MathType@MTEF@5@5@+=feaafiart1ev1aaatCvAUfKttLearuWrP9MDH5MBPbIqV92AaeXatLxBI9gBaebbnrfifHhDYfgasaacH8akY=wiFfYdH8Gipec8Eeeu0xXdbba9frFj0=OqFfea0dXdd9vqai=hGuQ8kuc9pgc9s8qqaq=dirpe0xb9q8qiLsFr0=vr0=vr0dc8meaabaqaciaacaGaaeqabaqabeGadaaakeaacqWGqbaudaqhaaWcbaGaem4zaCMaemiCaahabaGaeGymaedaaaaa@31B2@) and descriptors (e.g. Dgp1
 MathType@MTEF@5@5@+=feaafiart1ev1aaatCvAUfKttLearuWrP9MDH5MBPbIqV92AaeXatLxBI9gBaebbnrfifHhDYfgasaacH8akY=wiFfYdH8Gipec8Eeeu0xXdbba9frFj0=OqFfea0dXdd9vqai=hGuQ8kuc9pgc9s8qqaq=dirpe0xb9q8qiLsFr0=vr0=vr0dc8meaabaqaciaacaGaaeqabaqabeGadaaakeaacqWGebardaqhaaWcbaGaem4zaCMaemiCaahabaGaeGymaedaaaaa@319A@). Identical proteins from SwissProt and GenPept records, Psp1
 MathType@MTEF@5@5@+=feaafiart1ev1aaatCvAUfKttLearuWrP9MDH5MBPbIqV92AaeXatLxBI9gBaebbnrfifHhDYfgasaacH8akY=wiFfYdH8Gipec8Eeeu0xXdbba9frFj0=OqFfea0dXdd9vqai=hGuQ8kuc9pgc9s8qqaq=dirpe0xb9q8qiLsFr0=vr0=vr0dc8meaabaqaciaacaGaaeqabaqabeGadaaakeaacqWGqbaudaqhaaWcbaGaem4CamNaemiCaahabaGaeGymaedaaaaa@31CA@ and Pgp1
 MathType@MTEF@5@5@+=feaafiart1ev1aaatCvAUfKttLearuWrP9MDH5MBPbIqV92AaeXatLxBI9gBaebbnrfifHhDYfgasaacH8akY=wiFfYdH8Gipec8Eeeu0xXdbba9frFj0=OqFfea0dXdd9vqai=hGuQ8kuc9pgc9s8qqaq=dirpe0xb9q8qiLsFr0=vr0=vr0dc8meaabaqaciaacaGaaeqabaqabeGadaaakeaacqWGqbaudaqhaaWcbaGaem4zaCMaemiCaahabaGaeGymaedaaaaa@31B2@ respectively, are instantiated as a single non-redundant protein object *P*^1 ^on the graph. Similarly, Psp2
 MathType@MTEF@5@5@+=feaafiart1ev1aaatCvAUfKttLearuWrP9MDH5MBPbIqV92AaeXatLxBI9gBaebbnrfifHhDYfgasaacH8akY=wiFfYdH8Gipec8Eeeu0xXdbba9frFj0=OqFfea0dXdd9vqai=hGuQ8kuc9pgc9s8qqaq=dirpe0xb9q8qiLsFr0=vr0=vr0dc8meaabaqaciaacaGaaeqabaqabeGadaaakeaacqWGqbaudaqhaaWcbaGaem4CamNaemiCaahabaGaeGOmaidaaaaa@31CC@ and Pgp2
 MathType@MTEF@5@5@+=feaafiart1ev1aaatCvAUfKttLearuWrP9MDH5MBPbIqV92AaeXatLxBI9gBaebbnrfifHhDYfgasaacH8akY=wiFfYdH8Gipec8Eeeu0xXdbba9frFj0=OqFfea0dXdd9vqai=hGuQ8kuc9pgc9s8qqaq=dirpe0xb9q8qiLsFr0=vr0=vr0dc8meaabaqaciaacaGaaeqabaqabeGadaaakeaacqWGqbaudaqhaaWcbaGaem4zaCMaemiCaahabaGaeGOmaidaaaaa@31B4@ are mapped to a single *P*^2^. As a result, the two interaction objects Ibi1
 MathType@MTEF@5@5@+=feaafiart1ev1aaatCvAUfKttLearuWrP9MDH5MBPbIqV92AaeXatLxBI9gBaebbnrfifHhDYfgasaacH8akY=wiFfYdH8Gipec8Eeeu0xXdbba9frFj0=OqFfea0dXdd9vqai=hGuQ8kuc9pgc9s8qqaq=dirpe0xb9q8qiLsFr0=vr0=vr0dc8meaabaqaciaacaGaaeqabaqabeGadaaakeaacqWGjbqsdaqhaaWcbaGaemOyaiMaemyAaKgabaGaeGymaedaaaaa@318C@ (BIND) and Idi1
 MathType@MTEF@5@5@+=feaafiart1ev1aaatCvAUfKttLearuWrP9MDH5MBPbIqV92AaeXatLxBI9gBaebbnrfifHhDYfgasaacH8akY=wiFfYdH8Gipec8Eeeu0xXdbba9frFj0=OqFfea0dXdd9vqai=hGuQ8kuc9pgc9s8qqaq=dirpe0xb9q8qiLsFr0=vr0=vr0dc8meaabaqaciaacaGaaeqabaqabeGadaaakeaacqWGjbqsdaqhaaWcbaGaemizaqMaemyAaKgabaGaeGymaedaaaaa@3190@ (DIP) are mapped to the same object *I*^1^.

∑=∪DD∑=∪D∪d∈Dσ(d)
 MathType@MTEF@5@5@+=feaafiart1ev1aaatCvAUfKttLearuWrP9MDH5MBPbIqV92AaeXatLxBI9gBaebbnrfifHhDYfgasaacH8akY=wiFfYdH8Gipec8Eeeu0xXdbba9frFj0=OqFfea0dXdd9vqai=hGuQ8kuc9pgc9s8qqaq=dirpe0xb9q8qiLsFr0=vr0=vr0dc8meaabaqaciaacaGaaeqabaqabeGadaaakeaacqGHris5cqGH9aqpdaWeqbqaaiabdseaenaaBaaaleaacqGHris5aeqaaOGaeyypa0ZaambuaeaadaWeqbqaaGGaciab=n8aZjabcIcaOiabdsgaKjabcMcaPaWcbaGaemizaqMaeyicI4SaemiraqeabeqdcqWIQisvaaWcbaGaemiraqeabeqdcqWIQisvaaWcbaGaemiraqeabeqdcqWIQisvaaaa@4338@

where set operations over graphs act separately on the nodes and the edges of these subgraphs. For example, Fig. 5 demonstrates the union of several subgraphs obtained from several different sources.

### Semantic data matching and the identity problem

Since the source databases might highly overlap it is important to address the problem of data redundancy. Eliminating redundancy is relevant to data consistency as well as database efficiency, both in terms of the space-usage and computation time. This is important in operations such as protein alignments where maintaining redundant similarity information would come at great expense of computation and storage requirements. More importantly, it helps to corroborate and complete the information that is associated with the same physical entity by different sources, thus compiling a more comprehensive and accurate context for each entity.

Our data model is non-redundant in the sense that identical source documents (in their Biozon representation) are mapped to the same document node in ∑. Consequently, graph nodes of ∑ are frequently shared between sources. Formally, we say that two elements *d *∈ *D *and *d' *∈ *D' ***overlap **if σ(*d*) = (**v**, **e**) and σ(*d'*) = (**v'**, **e'**) and **v **n **v' **≠ ∅, i.e. there exists a document *v*_*i *_∈ **v **and a document *v*_*j *_∈ **v' **such that *v*_*i *_= *v*_*j*_. In the context of data integration and semantic data mapping, the concept of identity is particularly relevant to objects, and the key to our protocols for eliminating redundancy is the equivalence operators used and the reliance on objects in what we call an *object-centric model *(this is unlike most databases, where specific identifiers such as accession numbers are used to identify documents, not always uniquely). Specifically, two documents *v*_*i*_, *v*_*j *_are considered identical if *class*(*v*_*i*_) = *class*(*v*_*j*_) = *c *and *c *∈ **C**_*O *_and *object*(*v*_*i*_) = *object*(*v*_*j*_). The notion of identity depends on the object type, and for each class *c *we define an equality operator ≡_*c *_that is able to determine if two documents of that type are redundant (*v*_*i *_≡_*c *_*v*_*j*_). For example:

• For strings such as DNA sequences or protein sequences, a string match operator is used to determine identity.

• For sets of physical objects (e.g. interaction), the set-identity operator is used.

• For arbitrary subgraphs, graph isomorphism is used.

**Figure 6:**
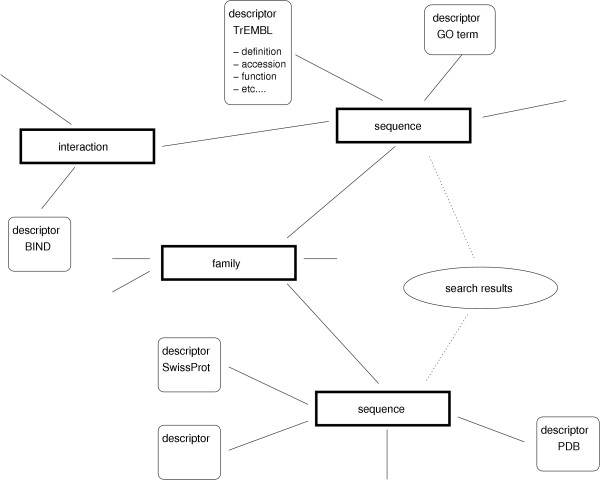
**A subset of the Biozon data graph**. Objects (rectangular shapes), descriptors (rounded boxes), and the relations between them form a typical subset of the Biozon data graph. The subgraph consists of two protein sequences that are described by a number of different descriptors and are related to a common Family object. Creating this graph requires data from a number of different databases or computations. Gathering data is a matter of traversing a portion of the graph and examining the nodes. For each node, it is possible to obtain a set of all relations connecting that document to another. Searches serve as an entry point to the data graph, from which the graph may be navigated to see the object's context.

This non-redundant implementation unambiguously and efficiently relates data sets together through shared objects. The outcome of such non-redundant integration is exemplified in Fig. 6. Our data integration protocols extend beyond eliminating redundancy between physical objects, and are applied in a more general form during updates (See 'Updates' section of the supplementary material) to compare all types of documents including descriptors.

From a computational standpoint, knowing the physical objects themselves is necessary, and is prerequisite for mapping them onto the Biozon data graph. Unfortunately, many types of source data do not include complete object definitions, and instead contain only references (e.g. DIP [5], InterPro [29]). To integrate such data into Biozon, it is necessary to map accession numbers to the physical objects they represent and create relations based upon that mapping. This necessity exposes many of the problems and uncertainties inherent in using accession numbers or arbitrary identifiers to represent concrete biological objects. For example, databases such as SwissProt and TrEMBL use accession numbers to refer to protein sequence entries. However, sequences might change and still retain their accession number. When the physical sequence data is used by others (such as the protein domain database InterPro) to derive meta-data, this becomes a major issue. Since there is no easy way of tracking the original sequence entries that were used to generate the meta-data, conflicts arise as meta-data that was associated with a specific sequence entry *X *may be mapped to a different sequence entry *Y *based on the accession number. To resolve that, InterPro introduces CRC64 checksum codes that serve to indicate that there is a conflict and a non-negligible number of these conflicts do occur. [InterPro provides data from a number of diverse sources such as PRINTS [30], ProDom [31] and Pfam [32] that identify regions of proteins that represent a particular domain or functional site. These regions are detected by matching the sequence against a particular signature, such as a regular expression or hidden Markov model. Each of the matching sequences are referred to by an accession number, and a 64 bit CRC value for the matching protein sequences is provided. Of the 4,727,510 mappings provided by InterPro version 8.0, 32,991 failed to match the corresponding proteins in SwissProt and TrEMBL versions 44.7 and 27.7 based on the CRC64 checksum codes. Most of these failures were due the fact that the InterPro mappings were created with respect to older versions of the SwissProt and TrEMBL databases. In the five months InterPro version 8.0 was active, SwissProt advanced from version 43.5 to 45.1, with similar advances in TrEMBL.] In response to these conflicts, Biozon employs various methods to map identifiers to concrete objects, including retrieval of archived entries or the use of CRC keys to search for possible matches, followed by comparison of the sequence entries. Because these results are materialized on the data graph, this operation needs to be performed only once at the onset of integration.

The direct impact of our model and data integration protocols is clear; It creates a single resource whereby relationships between objects are explicit and unambiguous. Eliminating object redundancy between diverse sources makes observations on their overlapping domains of knowledge efficient and programatically straightforward. For example, a total of more than 4,000,000 proteins from several databases (including SwissProt TrEMBL, PIR, GenPept, SCOP, PDB, DBJ, PATAA, PRF and REF) were reduced to a total of about 1.8 million sequences, after removing exact duplicates. Similarly, about 101,000 interactions that were derived from BIND, DIP and HPRD were unified into 76,000 unique interactions, using the set-identity operator [In BIND and DIP, interacting proteins are expressed in terms of identifiers to database entries in other databases. To identify redundancy in the interaction data set, records in these databases were mapped first to the physical sequences in Biozon.]

**Figure 7:**
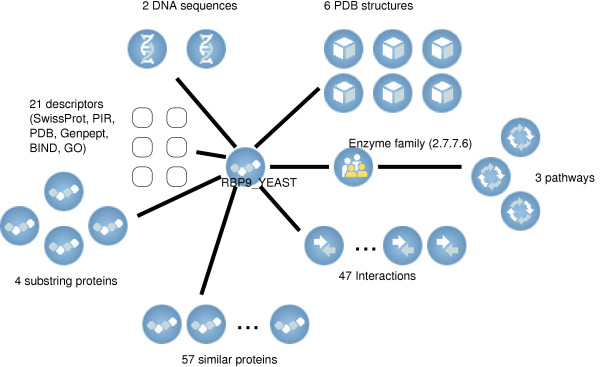
**The broader context of RPB9_YEAST as appears in Biozon**. DocID is 262161

An additional substantial benefit is that by integrating annotations from different source databases, an even more comprehensive resource of knowledge is created, since the accumulated information from several databases can compensate for missing information in others. This information is readily available from a single point of access in Biozon. For example, consider SwissProt :Q7RU07, which refers to a protein sequence with docID 363051 (We refer to entities using their unique and stable Biozon 'docID'. To view an entry with docID *x*, follow the URL http://biozon.org/Biozon/Profile/x). The definitions contributed by different sources vary, including 'Small membrane protein 1' and 'cervical cancer oncogene 9'. This protein is also defined in some records as 'hypothetical protein'. If one were to use these sources individually, the functional information present in others is missed. Moreover, data integration can also help to identify and resolve conflicting annotations between different databases, as is the case for SwissProt :ATPE_RICCN (docID 225475) that is assigned to two different enzyme families: EC 3.6.3.14 (by SwissProt) and EC 3.6.1.34 (by PIR and GenPept). Whether this is a typographical error or a fundamental difference in characterization is not known *a priori*, but both conflicting annotations are visible to the user. Most importantly, data integration serves to expose the broader biological context of an entity; information that can be very instrumental in functional analysis. For example, SwissProt RPB9_YEAST (DNA-directed RNA polymerase II) is linked to no less than 56 objects and 21 descriptors, as is depicted in Fig. 7. including interactions with other ribosomal proteins and tRNA molecules, structures of complexes involving this protein and the pathways of purine and pyrimidine metabolism. Data integration can also be useful in compiling missing information at the relation level. For example, to relate DNA sequences to their likely protein products we complement the information that is provided by NCBI for UniGene clusters by exploring other paths in the Biozon data graph that can be established between DNA sequences and proteins (e.g. through the 'substring' relation or through other members of UniGene clusters that can be mapped to proteins using the 'encodes' relation).

Despite all these advantages, it should be noted that from other viewpoints, entities of identical physical properties are not considered the same object. For example, even 100% identical protein sequences in different species might have different properties. Future versions of Biozon will allow one to view an entity in its organism-specific context, derived by projecting the data graph onto the organism of choice (see the section on Future Work in the Conclusion).

### Updates

Our choice of a tightly integrated schema with locally warehoused data was motivated by the many advantages that such a model possesses, as is exemplified throughout the paper. However, this choice has other consequences, as special maintenance protocols have to be designed to handle updates in the source data. These protocols must be designed so as to guarantee consistency within Biozon and between Biozon and the external sources. This is a difficult problem since the updates in external databases are not synchronized with one another. Moreover, the updates can potentially affect the status of derived data Since the biological objects in Biozon are non-redundant, we had to design methods to determine which operations in an update should be undertaken to achieve the desired effect without violating consistency. Besides considering consistency with regard to source databases, we had to consider staleness of derived data and initiate computations or delete derivations when appropriate. The Biozon schema was designed to address all these issues and our solutions are described in detail in the 'Updates' section of the Supplementary Material.

### Derived data

Biozon is more than a warehouse of existing data; it integrates unique derived data that is computed in-house. Several types of derived data currently exist in Biozon, from similarity data between objects to modules that expand existing data types based on inference, refine existing objects, or generate new data types obtained by processing existing data types and other derived data.

#### Similarity data and inference

The similarity relation is central to functional inference in biology. For example, the analysis of new genes usually starts with a database search, and their biological function is often predicted based on their sequence similarity with other, well-characterized genes. To maximize the utility and potential of computational functional inference, it is important to consider similarity relations over biological entities in addition to other explicit relations. These relations should be at the essence of any biological knowledge resource. This is true not only for proteins, but for other entities as well; one can think about similarity measures over protein families [33], pathways [34] or organisms [35].

Moreover, it is important to have access to multiple similarity indices, based on different measures. Consider proteins for example. Existing sequence comparison algorithms can be sensitive to the choice of parameters (e.g. the scoring function). Therefore, to detect homology, it is sometimes necessary to compare proteins using multiple sets of parameters. Furthermore, sequence-based measures often fail to recognize subtle similarities between sequences that have diverged greatly. In these cases it is necessary to use other methods of comparison based on structure, expression data or other attributes. However, obtaining these results requires access to algorithms that for the most part are not readily available and are too computationally intensive to be used on a daily basis. The need for a system that will store optimal results and accurate alignments based on multiple methods is even more evident as new, more sophisticated comparison algorithms emerge.

We address these issues by generating extensive similarity indices, based on a variety of comparison methods. Biozon currently includes similarity relations based on sequence (with more than 6 billion significant pairwise similarities), structure (with more than 8 million significant structural similarities), and similarities based on gene expression data. Other similarity measures will be gradually integrated into the system. [Certain similarities are computed using multiple algorithms. This is the case, for example, when comparing protein structures. As opposed to sequence similarity, there is no natural definition of structural similarity. Consequently many different algorithms were developed over the years, based on different approaches and definitions, producing results that can differ quite markedly. To address this problem we compute structural similarities for all PDB structures using three different algorithms: Structal [36], CE [37] and the in house URMS-RMS algorithm [38]. The results of all three algorithms are available at the Biozon website.]

The similarity data allows new and powerful modes of data querying and extrapolation as discussed in the 'Utility and Discussion' section. It enables propagation of information from well studied genes to other, less characterized genes, and facilitates fast transfer of knowledge to entities untouched by experimentation so far. For example, [TrEMBL: Q07992] (Biozon docID 272323) is an uncharacterized Yeast ORF protein (documented as an unnamed protein in GenPept and probable membrane protein in PIR). However, examination of proteins with similar expression profiles suggests that this protein possesses some ribosomal activity as it is strongly linked to other ribosomal proteins. Biozon contains numerous examples like that, of uncharacterized biological entities that can be partially categorized based on sequence, structure or expression similarity.

Beyond functional inference, similarity data is used to expand existing data types. For example, we are in process of constructing 3D models for proteins of unknown structures based on sequence homology with proteins of known structures (see [39]). We also use sequence similarity data to extend experimentally verified data sets on protein-protein interactions. Furthermore, we employ expression similarity to predict new relations between genes, such as common pathways or common promoter signatures, even when this information is not directly available [40]. To ensure data quality, such predictions are marked clearly, and users are provided with additional information (e.g. significance of homology) to help assess the validity of predictions (e.g. see Biozon profile of docID 109069957).

#### Data refinement

The data that is integrated into Biozon often overlaps, resulting in multiple descriptors that are associated with the same object. A synergistic approach that builds on this knowledge can often help to better characterize existing objects. This is the basis for modules of derived data that serve to refine instances of existing data. For example, all the descriptors that are associated with a protein sequence can be combined together to generate a more accurate or a more detailed description of the protein object (on average, 2.5 descriptors and definitions are associated with every protein object). Similarly, multiple descriptors can help assess the quality or increase the confidence in the existence of an object or a measurement, when the experimental protocols are noisy (as is the case for protein-protein interactions, many of which are determined by high-throughput techniques such as yeast two-hybrid that are not always reliable).

#### New data types

**Figure 8:**
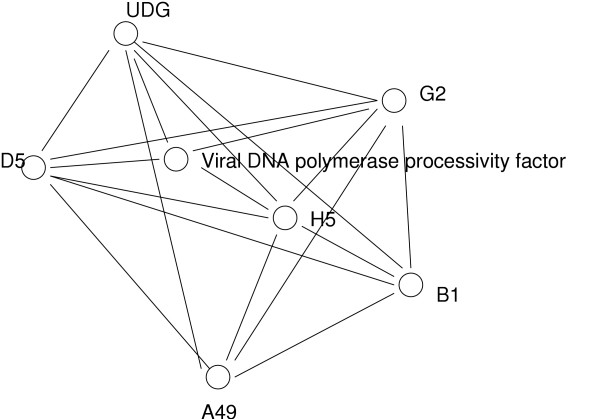
**An interaction map of Vaccinia virus proteins**. The protein-protein interaction data in Biozon can be viewed as a subgraph, with many interconnected elements. From this graph we compiled the set of all connected components, and each component was embedded in a two-dimensional Euclidean space, using the algorithm of 48 with the graph distances as input. The map shown is a subnetwork of Vaccinia virus proteins that seem to control its activity through a series of mediated interactions or by forming a complex. For example, the inactivation of protein G2 (docID 507266) renders the virus dependent upon isatin-beta-thiosemicarbazone for growth. This protein interacts with Envelope protein H5 (docID 465934) that interacts with protein A49 (840436) whose function is unknown, as well with Viral DNA polymerase processivity factor. The latter interacts with UDG (Uracil-DNA glycosylase docID 502617), as well as with protein D5 (Putative DNA replication factor). Proteins that directly interact are positioned closely in this map, while proteins that are connected through mediated interactions are positioned farther apart. The set of 7 proteins in this connected component form an interesting subgraph that was exposed with the embedding algorithm.

Biozon also introduces new relations or new data types that are generated by processing the source databases. For example, we use the 'encodes' relation, together with similarity data, UniGene clusters [41], and the 'substring' relation between DNA sequences to map human and mouse EST sequences to their protein products . As another example, we use the descriptors associated with protein sequences to associate proteins with Enzyme families, and a total of 156,276 proteins are classified into 3,944 families. Furthermore, the underlying graph structure of Biozon can be mined in itself to search for specific subnetworks of special interest. One such example is **interaction maps **that provide a bird's eye view of complex biological systems as is illustrated in Fig. 8. These maps are compiled from the interactions that are stored in Biozon.

### Utility and discussion

One of the major goals in Biozon's design is to provide means to effectively search and understand the data within it. The shape of the complete data graph is an emergent property and by utilizing its link structure we were able to develop and support new methods of query that improve data expressiveness in searches and representation.

### Complex searches

While all existing biological databases allow basic forms of search (e.g. of definitions, keywords, etc) they rarely allow one to search broad and complex biological contexts that span multiple data types. Specifically, it is difficult to form queries that search for interconnected sets. However, such queries are fundamental to computational and experimental studies in biology. Advanced cross-datasource search capabilities can be found in a few tools such as SRS [12], BioMediator [15], Columba [42], and DiscoveryLink [14]. Most achieve such capability by effectively creating joins between datasets based upon explicit cross references found when one source references another. Columba differs somewhat in that it also incorporates crosslinks between SwissProt protein sequences and PDB chains using similarity. Biozon offers a fundamentally different complex search mechanism that uses graph isomorphism to find patterns of related objects that match a given query that specifies *relationships *between objects in addition to their own inherent properties. The graph search space naturally contains all the edges on the Biozon graph, which are determined by previously mentioned data integration principles, not explicit cross references.

A complex query is comprised of a set of query nodes, a set of constraints specified on those nodes, and the edges connecting them. Queries, then, have a specified graph structure referred to as the **query graph**. Nodes of a query graph are labeled with the data type they represent, and any constraints that must be met for a match to occur (such as specifying a minimum length for an amino acid sequence or an EC number for an enzyme family). Executing these queries entails performing a search over ∑ for subgraphs of instances (**instance graphs**) that match the query. A match requires graph homomorphism, such that each node of the instance graph matches in type and constraints to the corresponding node in the query graph. Currently in Biozon, the matching instance graphs are projected on one of the query data types (referred to as the *query target*), as specified by the user, resulting in a non-redundant set of instances from that data type.

An example of a complex query would be: "human proteins that are members of an enzyme family that is part of a known pathway, and have a solved 3D structure". This query represents a graph with four nodes: Structures, Proteins, Enzyme families, and Pathways, and the query target is 'Proteins'. Edges are implicit in the sense that that are determined from the Biozon data graph. (One of the complications that arise when querying the Biozon data graph is that there are multiple ways to connect instances of different data types. This is addressed by introducing the notion of *Data topologies*, as discussed below.) Additionally, there is a constraint that the proteins must be human proteins. This query returns 105 results, out of 35261 structure to protein relations, 156276 protein to enzyme relations, and 2955 pathway to enzyme family relations. Another example would be "structures with resolution higher than two angstroms, of proteins that are in the 2, 3-butanediol dehydrogenase enzyme family". Such query involves the Structure, Protein, and Protein Family data types and specifies properties for two. In our current implementation, each data type and relation type is instantiated as a table in a relational database, and complex searches are translated into an SQL query that performs graph homomorphism by way of joins on the appropriate relations between interrelated objects. These joins operate on the non-redundant backbone of objects and use internal docID keys. Hence they are relatively simple to formulate in SQL and are efficient to execute (for more details see the 'Interface and Query' section in Supplementary Material).

### Fuzzy searches

The integration of similarity data into the Biozon schema allows for even more sophisticated methods of query. Specifically, Biozon uniquely extends queries to support *fuzzy relationships *by means of similarity. Fuzzy searches greatly increase the impact of data integration, since information is propagated from known objects that were studied experimentally, and were annotated extensively, to new objects with similar physical properties that await analysis. As such, fuzzy relations can make the difference between an uninformative search and a successful one.

Every similarity relation is associated with a significance or confidence value (e-value). This attribute can be specified in a fuzzy search, to limit the results to entities whose similarity exceeds a certain significance threshold. As an example, consider a simple fuzzy search over a single entity such as the protein with the SwissProt ID of 'DORS_DROME', an embryonic polarity dorsal protein in Drosophila. Initiating a fuzzy search for that protein with an e-value of 1e-100 returns 8 results that are similar to this protein within that threshold. Changing the threshold to 1e-50 includes weaker matches, extending the result count to 80. A fuzzy search over a single entity is equivalent in principle to a BLAST search with that protein as a query; but since we materialize the similarity relations from BLAST, this search is done almost instantaneously in Biozon.

However, the real power of fuzzy searches stems from the combination of similarity relations with our ability to search over sets. For example, one can search for the set of all proteins that have Stromelysin in their definition or are *similar *to any protein in this *set*. This query with a threshold of le-100 returns 81 records, and 379 records with an e-value threshold of 0.1 (as opposed to only 28 records when similarity relations are ignored). This query is equivalent to multiple BLAST searches over all proteins that have 'Stromelysin' in their definition, followed by unification of the results. Clearly, this would be a very time-consuming task if BLAST were to run in realtime over all these protein queries. However in Biozon this query is a straightforward generalization of a fuzzy search over a single entity and is almost as fast.

### Fuzzy complex queries

With the ability to produce similarity results from set input, a natural progression is to combine this ability with complex queries. A query or query part can be viewed as a set in and of itself. Consequently, similarity relations may be introduced at various points in a complex query. It is in this ability that the impact of similarity relationships on the results becomes immediately apparent, filling the gaps of incomplete information.

**Figure 9:**
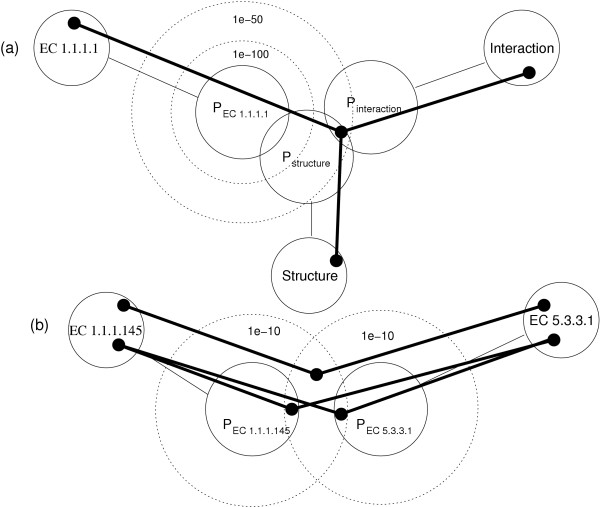
**Graphical representation of a fuzzy search**. (a) Complex searches find paths in the data graph. In this pictorial representation, nodes in result paths must occur where sets of objects satisfying different search constraints intersect. Introducing similarity extends some query steps to include similar results, thus enabling the discovery of paths in the graph where none existed before. This graph illustrates a complex fuzzy search for structures of proteins that belong to enzyme family 1.1.1.1 and are involved in known interactions. Circles on the graph represent sets of matching documents, and where they intersect, there are matches. The dotted lines represent extensions to the sets based on similarity. Without similarity, the set of proteins with structures (*P*_*structures*_) intersects with the set of proteins in enzyme family 1.1.1.1 (P_1.1.1.1_), meaning that there exists a protein with a structure that is a 1.1.1.1 enzyme. Likewise, *P*_*structure *_intersects with *P*_*interaction*_. However, there is no intersection between the three sets, and therefore no proteins that are in family 1.1.1.1 and involved in an interaction. Creating a fuzzy search with threshold of 1e-100 extends the set of 1.1.1.1 proteins but there are still no matching results. Increasing the threshold to 1e-50 produces the desired intersection, thus allowing connected paths spanning the entire query space. (b) Similarity may be introduced at multiple graph steps, further increasing the solution space to a complex query. For example, a search for E. Coli proteins that are members of enzyme families 1.1.1.145 and 5.3.3.1 returns no results. There are two possible areas in the query graph where similarity relations may be used to extend the query to fuzzy results: on proteins that are classified as 1.1.1.145, and on proteins that are classified as 5.3.3.1. When the evalue threshold is reduced to 1e-10 one protein (docID 737980) is returned with intriguing similarity to proteins that contain both domains. These proteins are observed in higher organisms as part of the estrogen, androgen and C21-Steroid hormone metabolism pathways.

As a simple example, suppose one is researching the structure of butanediol dehydrogenase enzymes (EC 1.1.1.4). A search on the PDB site on March 16 2005 returns no matching structures. However, although no protein with a defined structure has been mapped to that enzyme family, it is likely that there exists a similar, possibly homologous protein that does have a known structure, but has not yet been completely annotated and characterized as a member of the family. Indeed, a complex fuzzy search on the Biozon data graph for structures of proteins in the 1.1.1.4 enzyme family or of similar proteins returns a non-empty set. This complex fuzzy search first computes the set of proteins known to be in enzyme family 1.1.1.4. From there, it creates a larger set of every known protein that is *similar *to at least one in the 1.1.1.4 set. The query returns all structures that are related to proteins in this large, similarity-extended set. Using an e-value threshold of 1e-90 returns a single structure, oxidoreductase: cryatal structure analysis of meso-2, 3-butanediol dehydrogenase ([PDB:1GEG], biozon docID 8515367). As it turns out, this structure is of a protein that is highly similar to another protein (docID 550087) that is member of enzyme family 1.1.1.4. Furthermore, increasing the e-value threshold in the search to 1e-30 returns 5 matching structures, and 1e-20 has 53 and so on. The extension of sets based on similarity as part of fuzzy searches is demonstrated in Fig. 9a. Note that complex queries can have an arbitrary number of query nodes. Consequently it is possible to generate queries where similarity may be introduced at multiple junctures, producing different result sets (see Fig. 9b).

Finally, the similarity relation is not limited to sequence similarities between proteins. Biozon currently stores similarity relations between structures and similarities between genes based on expression-profiles.

With that data materialized, one can search, for example, for all proteins that are known to take part in a specific pathway, or proteins with similar expression profiles (associated with the corresponding mRNA sequences) to these proteins.

Because the validity of a given fuzzy search result depends greatly on the method of similarity employed (i.e. BLAST, yeast expression profile similarity), as well as parameters such as e-value, it is important to make the provenance of all matches available for inspection. In response, each search result that incorporates a fuzzy step is clearly marked in the results page. By clicking on the markings, the user is shown a representation of the exact instance tuple that includes every similarity step used, with corresponding links to the similarity data, such as a representation of a full Smith-Waterman alignment between two protein sequences.

It should be noted that many similarity relations happen to be local (e.g. multi-domain proteins might share only one domain in common). Therefore, not always it is possible to propagate information and draw conclusions based on similarity, as the functional features that are associated with the proteins might be localized to parts that are outside of the similar region. Biozon stores additional information on similarity relations that allows one to localize the relations. However, rarely is the case that functional features are localized and therefore it is currently difficult to take advantage of this capability of Biozon.

### Topologies

**Figure 10:**
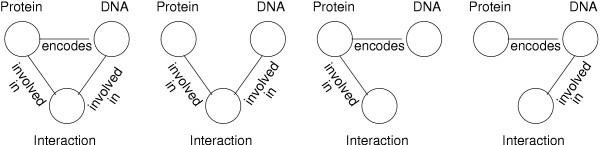
**Different topology graphs over the same data types**. These topologies involve the same three data types, but have completely different biological meanings. The first corresponds to a protein that is encoded by a DNA sequence and interact with it as well. The second indicates that the protein and the DNA sequence are interacting. The third indicates that the DNA encodes for the protein and the protein is involved in an interaction with a third partner, and the fourth indicates that the DNA sequence both encodes a protein and is involved in an interaction.

The Biozon graph is composed of many subnetworks with differing document compositions. The connectivity of the data graph has important consequences on searches, since there are multiple ways to connect instances of different data types. For example, proteins are connected to DNA directly as well as through interactions. Unless the query graph is explicitly specified, a query such as 'transcription factor proteins that are related to DNAs in humans' can be answered in many different ways, especially if considering paths that use other data types that are not specified explicitly in the query graph. Each path corresponds to a different set of instance graphs, and to obtain comprehensive results one has to consider all possible paths between the query data types. There are many issues involved with such queries, such as completeness and efficiency to name a few. More importantly, each path implies a different set of relations with a different biological meaning. Therefore, the meaning of an instance graph is as much dictated by the shape of the connected graph as by the contents of the documents within it. We refer to the graph shapes that occur within Biozon as *topologies *(Fig. 10).

**Figure 11:**
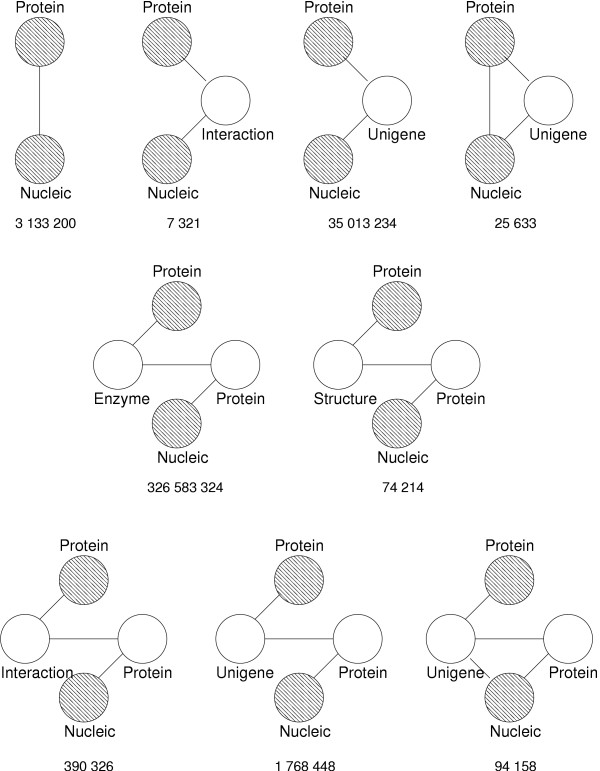
Observed graph topologies between proteins and nucleic acids with a maximum path length of 4. The number of occurrences of each topology instance is visible below each topology, using data current as of September 2005.

Beyond querying the emergent structure of the Biozon graph, topologies allow users to *discover *the emergent structure by characterizing the paths that relate objects together. For example, one may want to discover how a particular cancer related protein relates to known structures and interactions, or if any protein-protein interactions are involved in riboflavin metabolism. These can help discover previously unknown or unspecified relationships between known objects. For example, in Fig. 11 we show some of topologies that are observed when enumerating all possible paths of length 4 between proteins and DNA sequences (to view all such topologies, see ). These topologies reveal some interesting paths that cannot be detected by means of regular queries that query just direct relations. Since data topologies aim at detecting schema level graphs that are instantiated at the instance level they are difficult to query and process efficiently, and existing methods of query (such as traditional SQL or search systems such as Discover [43]) do not provide effective solutions. These and other issues are addressed in [44], where we have formalized the notion of data topologies in the context of heterogeneous (biological) data and present effective methods for querying topologies.

### Ranking biological objects

Organizing and sorting search results is an important part of information processing and extraction. The current status of biological databases resembles that of the pre-google days of the web. Existing methods for querying biological data that are available on the web generate lists of matches that are essentially random, or sorted based on features that are irrelevant to the query (for example, alphabetically). One would wish to have the results sorted based on their importance or relevance to the query. However, *a priori *it is not obvious how to quantify the importance of a match.

**Figure 12:**
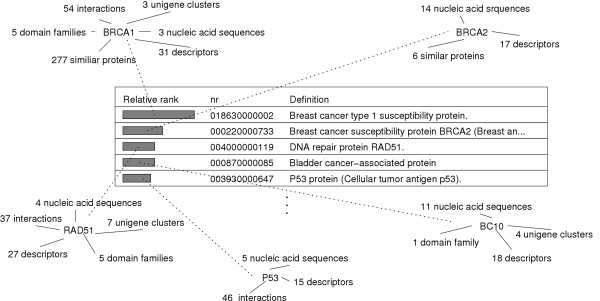
**Ranking of results**. These are the top 5 ranked results of a search for proteins with 'cancer' in their definition. Results of high rank tend to be linked to many other entities.

The underlying graph structure of Biozon is especially useful in that respect. By exploring this structure we can detect subgraphs of objects that are tightly interconnected. We view important or interesting instances in the result sets as those that are linked to many other important entities. This definition is motivated by one of our main goals: to provide users with the broader biological context of each individual entity. These subgraphs often share a common theme, and we refer to them as *Hubs of knowledge*. To detect these graphs and assign *prominence values *to the elements in the Biozon database we explored and tested quantitatively several spectral methods, including Hubs and Authorities [45], PageRank [46] and other models (the results of this study are described in detail in [47]). Our tests indicate that the PageRank method, similar to the method implemented in Google [46], is both more effective and more practical, compared to other models, and we have integrated into Biozon a ranking system which is based on that model. It should be noted that only the graph structure is taken into account when assigning ranks. Some data may possibly be viewed as inherently noisy or less reliable (take high throughput yeast two hybrid interactions, for example), others as immutable. These factors currently play no role in determining ranks in Biozon, though present an opportunity for future study (see [47]). As an example of the effectiveness of ranking, when searching Biozon for the query term 'cancer' we detect 1977 objects that match the term. A spectral analysis of this subgraph results in a ranking that returns as a top match a BRCA1 gene, Breast cancer susceptibility protein (docID 1079763). Examining the top five results as shown in Fig. 12 shows several other highly relevant proteins such as a p53 gene (docID 802537) that is related to multiple interactions and DNA sequences, all involved in tumor suppressing activity. This ranking utility has clear advantages over arbitrary orderings of result sets. For example, it can direct biologists that study specific genes or interactions to homologous genes or similar interactions in other organisms that are associated with more extensive experimental information, and expose them to knowledge that could have been overlooked otherwise.

### Implementation

This is a brief explanation of the current implementation of Biozon. For a more complete description, please see the section 'Implementation and practical implications' of the Supplementary Material.

### Hardware

The graph is managed by a single DBMS (PostgreSQL) that is resident on a Sun V880 server, with four 1.2 GHz UltraSPARC III CPUs and 8 Gb RAM. A 1TB of disk storage is used to store the Biozon graph and supporting data. A second backup server runs on a Sun V65x, with two 3 GHz Intel Pentium IV processors and 6 GB RAM, connected to a second 1TB array that contains a duplicated copy of the Biozon data graph. Large-scale computations and online analysis tools are run on a 50 node cluster comprised of Dell PowerEdge machines, with dual Pentium IV CPUs running at 1 or 2 GHz with 1 GB RAM each.

### Core database software

The biozon data graph is managed by the PostgreSQL DBMS (version 7.3). PostgreSQL was chosen because it is open source (BSD license) and has the extensibility and object-relational features that were necessary for developing Biozon. The schema is an exact analogue to the document and relation hierarchies (Fig. 2, 3) whereby each document or relation is instantiated as a table, and the hierarchy is implemented in PostgreSQL's inheritance model. Core data integrity, update, and data manipulation functions are written as triggers or stored procedures in C which are dynamically loaded at runtime (see Supplementary Material for a detailed discussion on these protocols).

Although not part of the DBMS itself, parsing and initial loading of data is done through custom scripts mostly written in Perl. These scripts are responsible for mostly straightforward translation of the data in its original form to a form suitable for loading directly into pre-loading tables in Biozon. The graph update algorithms are executed in the DBMS using functions written in C.

### User interface software

The user web interface is provided by a series of perl modules run with the Apache web server using mod_perl. The web interface is currently the only public mode of access to the Biozon data. Complex and fuzzy searches are implemented in this layer, whereby the user's query as built on the site is transformed into a suitable SQL query that is then executed by the DBMS. Currently, there is no external API or formal query language, though these are planned for a future release.

Public access to the source code to the interface is currently not available, as it is specific to our particular installation. Our primary focus has been in providing a service. Nevertheless, certain standalone components (such as some analysis tools) may be generally useful and therefore are available for download from the Biozon site. We also plan to make the schema and the core database software available.

### Analysis

There are essentially two modes of data analysis that Biozon currently performs. The first, large scale batch processing, occurs whenever the derived data in Biozon is updated, such as in generating our all vs all alignments or domain predictions. These jobs are launched across our entire compute cluster of 50 nodes and may take days or weeks to complete, depending on the nature of the computation and the amount of data being processed. Single-use analysis, as provided in the 'Analysis Tools' section of Biozon  are run on demand on user-provided data. Submitted jobs are scheduled on the least loaded cluster machines, and where applicable, comparison is performed with respect to the current version of the Biozon database. The software used in these analyses are available for download in the 'software downloads' section of Biozon ;

## Conclusion

We describe a system (Biozon) that unifies multiple data types from multiple resources into a single knowledge resource. Our system is based on a flexible non-redundant graph model, unambiguous representation of biological entities that relies on their physical properties, and maintenance protocols that are based on a set of modular authorities. Combined all together, these elements serve to complete and corroborate data, to detect conflicts between source databases, and most importantly, to expose the broad biological context of each entity. Data archiving is addressed through the use of time stamps. Thus data can be reproduced, browsed and materialized as of arbitrary time points in the past. Most importantly, the Biozon system was designed such that the biological context itself can be efficiently searched against and assessed. The intricate link structure of the data graph enables complex queries that span multiple data types, fuzzy searches that utilize the many similarity relations in Biozon, and a ranking system that is unique in the biological knowledge domain. The combination of these features is a first-of-kind in this field.

The amount of biological data available is increasing rapidly, especially due to the ongoing genome projects of human and other organisms. The logical schema and data model of Biozon was designed to accommodate this expected expansion and to allow easy integration of other data types and future databases by extending the existing document hierarchy. Moreover, the database infrastructure was designed to be easily maintainable, using update protocols that work to preserve consistency, both internal and external. Our graph schema, the document class hierarchy and the relation class hierarchy are based on physical, semantic, or logical differences between the types of data represented in Biozon. That being said, the structure of the schema and the hierarchies is not immutable; it is a design choice that balances the semantic requirements of the data in the source databases with current conventional wisdom. Our design choices are sometimes subjective and motivated by data availability, clarity and applications. As the data set grows and as more knowledge accumulates, this model can be expected to expand and change.

Beyond the development of advanced web based tools to support complex and fuzzy searches, we also attempt to channel the information directly from the source databases to the end users. Since the outcome of one's research is the input for another, users these days often want direct access to search results and are interested in downloading the data for further analysis. However, this is difficult for complex queries that span multiple data types as it requires access to multiple databases. Furthermore, generating interconnected data sets would require certain data manipulation expertise and might take days or weeks, depending on the user experience. Biozon's solution utilizes the user account system, allowing users to materialize and download the results of their queries. Moreover, since each document is associated with a timeline users can re-materialize the results as of arbitrary times in the past. This is especially useful if one is interested in reproducing the same dataset that was obtained when a research project was initiated based on the results of a certain query.

Biozon's user accounts serve additional purpose, as another channel for data dissemination. In most cases the data that is stored in databases is partial as it is extracted only from published literature. However, even after discoveries are made it might take years until the knowledge is stored in databases, and most of the information is actually out there, intellectually held be individuals who study closely specific biological entities. As a response, Biozon enables researchers to submit and deposit comments on specific genes, protein families, interactions or pathways, in the 'expert comments' section.

Finally, Biozon strives to make the knowledge stored within readily available to the whole scientific community, and gradually also the means for others to deposit, integrate and share their data. The Biozon database is accompanied with a sophisticated web interface where source data and computed data, and data analysis tools converge into a single working environment, online at .

### Future work

As Biozon continues to grow, a major focus on future effort will be in keeping the Biozon data up to date and incorporating new datasets. Currently, we update major databases once every few months and are gradually working towards more frequent and automated updates as resources allow. New datasets are regularly added to Biozon to fulfill specific needs or to create a more comprehensive list of the types of data represented (see release notes at ). Maintenance and expansion of data will always be a constant goal. Based on user feedback and opportunities presented in exploration of the graph structure, we have identified several areas for future research or development beyond maintenance of data, and we make brief mention of a few of them.

#### • General contextual views of the Biozon graph

We intend to allow users to specify the context in which the graph will be searched and browsed (e.g. a specific organism, tissue, cell or subcellular location). Graph searching and browsing would be limited to a subset of the Biozon graph that is deemed relevant in a given context. For example, because proteins are unified based only on sequence their profile page will show all information relating to that sequence, regardless of species. A possible outcome is that a linked interaction that is present in species A but not species B will be linked to identical proteins from species B. Viewing the entry and searching the graph in the context of species B would ignore all information that does not pertain to that particular species. Work must be done to determine the contexts that users may be interested in, and developing tools to automatically project the graph on the relevant context or filter each graph operation by the desired context.

#### • Queries based on uploaded data

Queries would incorporate a user-provided dataset that would be used as a query node in a complex query. For example, consider the query *"Find all structures of proteins that interact with proteins in the set ***S***" *where **S **is a set of user data.

#### • Public access and API

Currently, the only public access to the Biozon database is through its web interface. While the materialization option gives users the ability to download the results of a query for possible further analysis, there is no general purpose API that would allow for the creation of third-party software modules that interface with the Biozon query engine or its graph directly. We plan to provide such access, but development in that regard has not yet started.

#### • Topology queries and display

As it stands now, search results are returned as a list of matching "target objects" that satisfy a given query, where the search topology is spelled out explicitly. Each target object is an instances of the specified topology. One particularly interesting idea is to create a search interface whereby individual graph elements are specified in the query, and the results are a set of topology instances that relate the graph elements together. In other words, this mode of search would discover paths in the graph between specified objects.

### Availability and requirements

Biozon can be accessed online at . Browsing of data, searching, and data analysis is accessible to all users, though more advanced features such as saving queries and commenting on objects require the user to create an account. In order to effectively use Biozon, a contemporary javascript-enabled browser is required. The data itself is copyright to their original publishers, and use granted under the terms set forth by each individual data source.

## Authors' contributions

GY leads the Biozon project and devised the object representation and integration principles. GY and AB co-designed the logical schema of Biozon and the front-end web interface. AB designed and implemented the Postgres-based Biozon schema and search engine, wrote the parsing and upload scripts and designed the consistency and update protocols. GY devised the graph formalism, and produced the derived data and analysis tools. Both authors wrote the manuscript.

## Supplementary Material

Additional File 1 **Supplementary material **This document contains more detailed information on several aspects of the Biozon system that are not discussed in this paper, such as data maintenance protocols, physical implementation and design and query formation. We also include a longer discussion on related studies. Click here for file
