# Supplementary material for: BIOZON: a system for unification, management and analysis of heterogeneous biological data
Source: BMC Bioinformatics. 2006 Feb 15;7:70. doi: 10.1186/1471-2105-7-70 (PMC1449871; doi:10.1186/1471-2105-7-70)
Supplement: Additional File 1 — Supplementary material This document contains more detailed information on several aspects of the Biozon system that are not discussed in this paper, such as data maintenance protocols, physical implementation and design and query formation. We also include a longer discussion on related studies. [file 1471-2105-7-70-S1.pdf]

Supplementary material for *BIOZON: a system for unification, management and analysis of heterogeneous biological data*

Aaron Birkland and Golan Yona*
Department of Computer Science
Cornell University, Ithaca, NY 14853

In this document we elaborate on several aspects of the Biozon system that are not discussed in the paper (BMC Bioinformatics), such as data maintenance protocols, physical implementation and design and query formation. We also include a longer discussion on related studies.

Contents

1 Implementation and practical implications **2**
1.1 Physical Structure . . . 2
1.2 Interface and Query . . . 3

2 Updates **5**
2.1 Consistency . . . 5
2.2 Handling updates . . . 6
2.3 The synchronization protocols: maintaining external consistency . . . 6
2.4 The primitive functions: maintaining internal consistency . . . 8
2.5 Effect of updates on derived data . . . 11
2.6 Consequences of updates with asynchronous sources . . . 13
2.7 Time context . . . 15
2.8 Implementation . . . 15
2.9 Analysis of a test case . . . 16

3 Related studies **17**
3.1 Mediated solutions . . . 18
3.1.1 Mediated layer . . . 18
3.1.2 Mediated schema . . . 18
3.1.3 Drawbacks of mediated solutions . . . 19
3.2 Warehouse solutions . . . 20

*Corresponding author: golan@cs.cornell.edu

1 Implementation and practical implications

We chose to implememnt our data model using the the PostgreSQL ORDBMS. This open source solution offered a flexible platform to implement our data model and application logic while maintaining adequate performance.

1.1 Physical Structure

We create a logical mapping between elements in our data model to its analogue in a relational database. Classes in the document or relation hierarchies map to a single table in the database, and these tables contain a set of columns that directly parallel the attributes defined for each class. Members of these classes are instantiated as individual records in their analogous tables. As an object-relational database, PostgreSQL offers inheritance of relations. We create an inheritance hierarchy parallel to the hierarchies present in our data model. Thus, A SELECT operation on a given table representing a class returns results that are a member of that class and any of the subclasses that inherit from it.

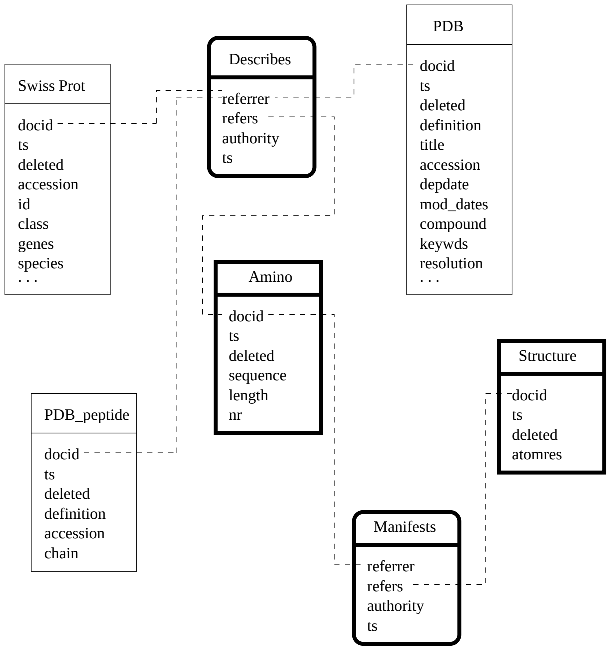

Figure 1: **Schematic schema representation**. A small subset of the Biozon relational schema showing a few connected objects, descriptors, and relations.

The root 'document' and 'relation' classes contain as attributes the minimal set required to support the methods of data consistency and time-stamping. As such, the document class consists of a globally unique docID identifier, a Boolean flag representing delete attempts, and an abstract data type (ADT) defined to represent the timeline. Relations consist of a pair of attributes 'referring' and 'referred' that represent the docIDs of the documents they relate as well as a timeline, and a field that indicates authority association (see section 2.4). Figure 1 shows an example of part of the detailed relational schema present in Biozon.

As is evident from figure 1, the high level graph structure is normalized, and joins between documents and relations are performed on the 'docID' and appropriate 'referring' or 'referred' field. What may not be so obvious is that most descriptor representation of external databases, such as SwissProt is not normalised.

Currently, we have adopted a simplified representation in this first phase of Biozon whereby the flat textual representation of data as represented in sources such as SwissProt is divided into a number of attributes of type ‘text’ instead of expanded to multiple tables and normalised. For example, the ‘refs’ field of our SwissProt descriptor is analagous to the portion of a SwissProt flat file that lists a set of publications that pertain to the described protein sequence. In Biozon, this field is represented as a single block of text rather than divided into separate entries (journal articles) and represented in a separate table. This implementation choice was chosen in the interest time and is tangential to the core data model. Eventually, we plan on adopting a more normalized representation for all descriptor annotation.

1.2 Interface and Query

The primary interface to Biozon is currently through the online browsing and querying tools. These interfaces form a layer that is intuitive to the non-programmer. The complex search interface, for example, provides a high level representation of the object graph and associated descriptor attributes that is oriented toward the non-programmer and hides all the implementation details of the schema.

Queries, as submitted by the user, are translated by the Biozon backend into the appropriate query SQL. This process is rather straightforward in that the joins necessary for complex queries are always on the docID field of documents and the referring or referred field of relations. The query generator has access to metadata describing the structure of the graph and the mapping between graph objects and physical table names. Likewise, metadata describes where particular attributes are defined in descriptors. These descriptors are joined with the objects that comprise the query.

The initial SQL query that is issued when generating a complex query is structured such that top level targets correspond to subqueries, each one returning records that represent instances of a single object type (directly analogous to a query node in a complex query graph). Also included in the top level targets of the initial query are all graph relations that connect the nodes. The WHERE clause serves to join the subquery objects with the appropriate edges to specify the exact paths searched on the graph

As an illustrative example, consider a complex search for *Structures with resolution less than 2 angstroms of proteins that are in enzyme family 1.1.1.1.* The raw SQL created by the query generator is as follows:

```
SELECT o_1.docid
 FROM
   (SELECT structures.docid AS docid
     FROM  PDB,
      describes AS pdb_describes,
      structures
     WHERE pdb_describes.referrer
           = pdb.docid
     AND pdb_describes.refers
            = structures.docid
      AND resolution <= '2'
      AND 'now'::date @ pdb.ts
      AND 'now'::date @ pdb_describes.ts
      AND structures.ts @< 'now'::date
   ) AS o_1,
   (SELECT amino.docid AS docid
     FROM amino
     WHERE amino.ts @< 'now'::date
   ) as o_2,
   (SELECT ec.docid AS docid
    FROM ec
    WHERE ec = '1.1.1.1'
```

```
23:    AND ec.ts @< 'now'::date
24:   ) AS o_3,
25:   manifests AS r_1_2,
26:   enzyme AS r_2_3
27:
28:  WHERE o_1.docid = r_1_2.refers
29:   AND o_2.docid = r_1_2.referrer
30:   AND 'now'::date @ r_1_2.ts
31:   AND o_2.docid = r_2_3.refers
32:   AND o_3.docid = r_2_3.referrer
33:   AND 'now'::date @ r_2_3.ts
```

Looking at this query, we observe that there are three top level subqueries aliased as `o_1`, `o_2`, and `o_3`. These subqueries return records containing at least the 'docid' column, and represent the Biozon objects Structures, Protein Sequences, and Enzyme Families, respectively. Additionally, we see targets of `manifests` aliased as `r_1_2` and `enzyme` aliased as `r_2_3`. These represent relations in our graph model. 'Manifests' is the relationship between a protein sequence and a structure and 'enzyme' is a subclass of 'contains', and is a relationship between an enzyme family and a protein sequence. In the `WHERE` clause of the query (lines 28–33), we see some rather straightforward join constraints that relate the objects together trough the appropriate relation to create a path in the graph $Structure \xleftarrow[manifest]{} Protein \xleftarrow[contain]{} Enzyme$

Digging deeper into the query, we see that lines (16–19) create the subquery `o_2`, which is comprised of the docIDs of all protein sequences. There are no constraints on the properties of the proteins themselves in the query. (20–24) define the set of enzyme families as `o_3` with the constraint that the 'ec' field, representing the family's EC number, is 1.1.1.1. This field is an attribute of the ec table.

Lines (3–15) are slightly less straightforward. Logically, `o_1` is made to be all structures with resolution less than 2 angstroms. Resolution, however, is not an attribute of a structure object directly. It is an attribute of a PDB document that *describes* the structure. Therefore, the `o_1` subquery, contains a join between `structures`, `describes`, and `pdb` such that all structures have a describes relationship with a PDB document that indicates a resolution of 2 angstroms. Since objects only contain attributes that are physically or logically defining, most constraints on annotation, such as definition, accession number, etc are actually placed on related descriptors. These are folded into the appropriate subqueries that return only records that satisfy all constraints.

Written in this manner, creating complex queries is relatively straightforward, and the code can be modularized into independent units, each building on their own part of the query before it is stitched together using only high-level graph structure metadata which indicates how high level objects are related on the graph.

After the query is generated, it passes through a re-write step where the query may be modified. Possible modifications include the addition of SELECT targets for accounting purposes and merging of query steps for the purpose of efficiency. PostgreSQL provides cost-based query optimization for selection of the optimal execution plan. The example given typically takes about 20 to 100 milliseconds to execute depending on the status of the buffer cache, system activity, etc.

Versioning is provided by operators on a 'timeline' ADT that indicates the visible lifetime of a particular graph element (see section 2.7). Line 33 of the query gives a typical example of how these are exploited. The `@` operator and its commutator `@<` are defined to compare a timeline with a date, returning 'true' if the date falls within the bounds of the timeline. As is evident in the example, including the appropriate versioning constraints in the query will produce results only valid in a snapshot at the given date.

2 Updates

A model that is based on a tightly integrated schema with locally warehoused and non-redundant data has several advantages as discussed in previous sections. However, the cost of having such a model comes in increased maintenance and consistency issues that emerge when the source data change, since we are charged with the task of updating our data frequently to reflect these modifications[1]. The updates in external databases are not synchronized with one another, and such dynamic data could potentially be involved in a set of locally derived relations that have multiple dependencies from several different databases.

Furthermore, biological knowledge in the constituent databases changes so rapidly, these changes might outpace analysis on a particular set of data that existed at a given point in time. In response, we require that data can be viewed as it existed at an arbitrary point in time in the past. Therefore, elements of the graph must be visible only within well-defined time contexts. Moreover, any operation to the graph in the present must not affect any snapshot of the graph in the past, and the effect of an operation performed at some point in the past must be visible in subsequent time contexts. This implies that no operation to the graph ever removes data, it can only add.

The Biozon schema was designed to address all these issues, so as to benefit from the all advantages associated with a tight schema mentioned above. We first present our definition of consistency and then the protocols used to maintain consistency under updates.

2.1 Consistency

We define consistency of objects in the database in terms of how they reflect their published counterparts and the derived data, and how they are depended on by others in the database. A fundamental principle is that data in Biozon must never contradict its source, and data must always be **externally and internally consistent**. We distinguish between two general types of consistency: knowledge consistency and structural consistency.

Knowledge consistency is concerned with descriptions of the same entity from different sources and differences in substantive content within the same domain of knowledge. Biozon strives to assure that its internal representation of a source database is an accurate reflection of the contents of that source. However, Biozon does not have control over the source data and therefore cannot guarantee its quality. Consequently, conflicts *between sources* can and do exist, and these conflicts are visible. For example, two databases may ascribe conflicting functions to the same protein, or give them substantially different definitions. As a less severe example, different databases may associate different features with a protein or cite different experimental evidence for an interaction. This latter example is common and even beneficial, where the former represents a genuine problem that domain experts would have to resolve. Biozon does not attempt resolving such conflicts, reporting such results just as they appear, however, it can help in detecting these discrepancies. As such, Biozon serves as a multi-expert system where the user is charged with the task of interpreting the data presented.

Structural consistency is concerned with the manifestation of the source data in Biozon. We define two types of structural consistency: internal and external. External consistency specifies the desired relation between elements of our database with respect to external databases. Let $\Sigma = (\mathbf{D}, \mathbf{R})$ denotes the unified database. Let D denote an external source database. Let D_v be some external database D as it appeared at time v, and D_u be D as it appeared at time u where $u < v$. The database Σ is said to be **consistent** with a particular version v of database D when

$$d \in D_v \rightarrow \sigma(d) \subset \Sigma$$

[1]It should be noted that similar issues exist when integrating data using a mediated schema.

and for every previous version u of D

$$d \in D_u \wedge d \notin D_v \rightarrow \sigma(d) \not\subset \Sigma$$

In other words, every element of the external database must be instantiated exactly in the Biozon data graph following its transformation function, and every element that was deleted cannot exist in its *entirety* in Biozon[2].

Finally, we define internal structural consistency based on the dependency between internal elements of Biozon. There are two aspects to internal consistency. First, we enforce graph consistency in terms of relationships between objects (similar to a foreign key constraint). If an object is referenced in a relation, internal consistency would guarantee the existence of the referenced object. In a non-redundant data model where relations are explicit and refer to objects that may be modified or deleted by any database of which they are a member, the need for this level of consistency becomes apparent. Second, we maintain functional consistency of the derived data with the elements used to derive it. Thus, there are two parts to the formal definition of internal consistency. Σ is *internally consistent* if

1. $\forall e = (v_1, v_2), e \in \Sigma \Rightarrow v_1 \in \Sigma \wedge v_2 \in \Sigma$
2. If $\sigma_d \in \Sigma$ represents derived data such that $\sigma_d = f(\sigma)$ for some σ then $\sigma \subset \Sigma$.

where $f(\sigma)$ is some function with subgraph σ as input that produces σ_d as its output. In simple terms (1) means that for every relation in the Biozon graph, both objects referred to by the relation must exist. Condition (2) is concerned with derived data in Σ. If Σ changes such that the derivation no longer holds true, the derived graph can no longer exist in its entirety.

2.2 Handling updates

There are three elementary operations on the graph nodes and edges that can comprise an update to Biozon data: addition, deletion, and modification. An update, for example, would change the value of attributes of a node, and delete may remove an edge from the graph. These operations are invoked when synchronizing Biozon with an external database. Wherever internal representations (in Σ) of different external database elements share graph nodes, maintenance of consistency, as defined above, deserves the utmost attention. We define low-level protocols of the elementary update operations that operate on the graph nodes and edges to enforce internal consistency. These protocols lie underneath higher level operations that perform periodic synchronization to bring D_Σ up to date with D. All in all, updates in Biozon are a three-level process: synchronization protocols that call high-level functions that operate on subgraphs (denoted by $Add()$, $Delete()$ and $Modify()$), and these in turn invoke low-level functions that operate on graph nodes and edges (denoted by $add()$, $delete()$ and $modify()$). In this section we start with describing the synchronization protocols and the high-level procedures, and continue to discuss the lower-level procedures in the next section.

2.3 The synchronization protocols: maintaining external consistency

The goal of the following protocols is to maintain external structural consistency with a source database in our non-redundant object model. Consider an external database D. Any element d in this database is mapped to a specific subgraph $\sigma(d) \subset \Sigma$ using the transformation function T_D such that $T_D(d) = \sigma(d) = (\mathbf{v}, \mathbf{e})$. Every element d of D is distinct. We denote the source key of d by $K(d)$, which is some set information or

[2]The element d is mapped to a set of documents and relations in Biozon $\sigma(d) = (\mathbf{v}, \mathbf{e})$. As will become apparent, even if an element was deleted, some of its documents or relations might still be part of the Biozon graph if they are part of subgraphs corresponding to elements from other sources that have not been deleted. However, there is no situation where the subgraph $\sigma(d)$ in its entirety is shared with other sources, as at least one document $v_i \in \mathbf{v}$ is unique to the source D.

attributes that serve to identify d uniquely[3]. In the Biozon implementation, the source key is instantiated as attributes in one or more documents in $\sigma(d)$, per the transformation function $T_D(d)$. To check if a specific source key exists in Biozon is a matter of searching for these attribute values in the corresponding document types. These attribute values can be viewed as the key of both the source element d and the corresponding graph $\sigma(d)$ such that $K(\sigma(d)) = K(d)$.

Assume Biozon contains a version u of D and denote by $\mathbf{K}(D_u)$ the set of all keys in version u of database D. Given a new version v of D, an update that will synchronize Biozon with D entails the following high level operations on the graph:

- For every $d \in D_u$ s.t. $K(e) \notin \mathbf{K}(D_v)$ consider d as a deleted element and issue $Delete(\sigma(d), \Sigma)$.
- For every $d \in D_v$ s.t. $K(d) \notin \mathbf{K}(D_u)$ consider d as a new element and issue $Add(\sigma(d), \Sigma)$.
- For every $d \in D_v$ s.t. $K(d) \in \mathbf{K}(D_u)$ consider d as a putative modification[4]. and issue $Modify(\sigma(d), \Sigma)$.

Since the subgraph $\sigma(d)$ is comprised of a number of nodes and edges, each high-level operation translates to several low-level operations. To delete $\sigma(d)$, means deleting every graph edge or node in the subgraph. Likewise, to add $\sigma(d)$ to the graph would entail adding all the corresponding nodes and edges. However, since we adopted a non-redundant object model, graph nodes can be highly connected and linked to documents from other external databases, and thus have many dependencies associated with them. Consequently, adding or deleting a subgraph is not a straightforward operation. For example, upon addition, the elements of $\sigma(d)$ must be first checked for redundancy with existing elements in Σ, using the appropriate identity operators as was described in section 'Integration' (in the main text), and if a match is detected, all the associated relations must be redirected as is detailed below. Deleting a document in Biozon is significantly more involved than addition in enforcing internal consistency in a non-redundant model. This complexity, however, is handled entirely by the low level protocols. The high level Delete protocol, then, is relatively simple. High-level operations $Add()$, $Delete()$ and $Modify()$ call the low-level functions $add()$, $delete()$ and $modify()$ and these in turn trigger a sequence of processes that take care of the relations pertinent to consistency or data propagation.

Let $\sigma(d) = (\mathbf{v}, \mathbf{e})$ and $\Sigma = (\mathbf{V}, \mathbf{E})$. Denote by $\mathbf{e}_v$ the set of relations that are associated with document $v \in \mathbf{v}$. Let $class(v)$ be the class of document v and let $\mathbf{V}_c = \{v \in \mathbf{V} | class(v) = c\}$. The exact procedure for each high-level function is described next.

$\underline{Add(\sigma, \Sigma)}$

1. For every document $v \in \mathbf{v}$, let $c = class(v)$
   (a) If $v \equiv_c u$ for some $u \in \mathbf{V}_c$ then for every relation $e \in \mathbf{e}_v$ such that $e = (v, w)$ replace e with $e' = (u, w)$. Similarly if $e = (w, v)$ then replace e with $e' = (w, u)$.
   (b) Else $add(v, \mathbf{V})$.
2. For every relation $e \in \mathbf{e}$ call $add(e, \mathbf{E})$.

As is evident from the high level protocol, step 1 matches documents in σ with their identical counterpart in Σ as determined by $\equiv_c$. Whenever a match is found, the candidate object v in σ is not added to the graph

[3]The source key can be as simple as an accession number (as is the case for SwissProt) or a complex set of attributes. For example, to identify uniquely an InterPro element we use the combined value of InterPro ID, protein ID, and coordinates.

[4]Note that even if the key has not changed, some aspects of the element d might have changed. Therefore we consider such elements as putative modifications.

and all relations in σ are modified to point to the matching database object u on Σ instead of v. Hence, step 1 prevents adding redundant documents[5]

Note that in step 1, v is any document, therefore it may be an object *or* a descriptor. That fact would imply that non-redundancy applies to descriptors as well, even though we only claim to have a non-redundant object model. In practice, most descriptors are collections of attributes with varying annotation and unique accession numbers pertaining to a single entity, and are therefore inherently distinct. For example, one never observes the same *identical* record appear more than once in the SwissProt database. Likewise, since SwissProt documents in Biozon simply mirror the annotation in SwissProt and are only associated with a single protein sequence, one will never observe two identical SwissProt descriptors in Biozon, and each SwissProt descriptor will be related to exactly one protein. However, to reduce redundancy even more and increase expressiveness, we have started implementing a modular approach towards descriptor representation that breaks up the information from a single external entity into multiple descriptors, one for each attribute-value pair. This approach is already employed to represent information on domain families, such as the data that originates from InterPro. Under that representation, a descriptor may be associated with multiple objects, and non-redundancy should be addressed. The protocols described above will enforce non-redundancy in such cases, as long as an identity operator is defined for these descriptors.

$\underline{Delete(\sigma, \Sigma)}$

1. For every document $v \in \mathbf{v}$ call $delete(v, \mathbf{V})$.
2. For every relation $e \in \mathbf{e}$ call $delete(e, \mathbf{E})$.

This high level protocol for delete is very simple. However, it is much more involved at the primitive level, as is explained in section 2.4.

$\underline{Modify(\sigma, \Sigma)}$

Since the key $K(d)$ exists in Biozon, then there exists a subgraph $\sigma' \in \Sigma$, $\sigma' = (\mathbf{v}', \mathbf{e}')$ such that $K(\sigma') = K(d)$ and there is one to one correspondence on nodes and edges with σ.

1. For every document $v \in \mathbf{v}$, denote $v' \in \mathbf{v}'$ the corresponding node in σ' and let $c = class(v) = class(v')$ If $v \equiv_c v'$ then do nothing.
2. Else call $modify(v', v, \mathbf{V})$ and for every relation $e' \in \mathbf{e}'_{v'}$ such that $e' = (v', w)$ replace e' with $e = (v, w)$, i.e. call $modify(e', e, \mathbf{E})$. Similarly if $e' = (w, v')$ then replace e' with $e = (w, v)$.

2.4 The primitive functions: maintaining internal consistency

The high level operations $Add(\sigma, \Sigma)$, $Delete(\sigma, \Sigma)$ and $Modify(\sigma, \Sigma)$ execute such that external consistency between D_Σ and D is preserved, but do not consider consistency relative to the internal representation of other databases D' in cases where D_Σ and D'_Σ overlap. Moreover, these operations might affect the consistency of the graph with respect to derived data. As opposed to the function $Add(\sigma, \Sigma)$, the functions $add(v, \mathbf{V})$ and $add(e, \mathbf{E})$ are the low-level functions that operate directly on the graph elements, as described in this section. The internal consistency, then, is provided by the low-level protocols on add, delete, and modify to **graph primitives** (nodes and edges). The combination of the the high-level and the low-level procedures leads to enforcing both internal and external consistency.

[5]The SQL implementation (see Section 1) of the low level add() operation will report a failure and abort the transaction if there is an attempt to insert a redundant object. This is implemented using a unique constraint or a defined check constraint.

Maintaining external and internal consistency requires considering the relations of each document to other documents and verifying that meaningful and valid relations between documents are not removed. For example, if the same sequence is defined in SwissProt and PIR, there would be a single sequence document representing the sequence, and at least two descriptor documents containing the PIR and SwissProt annotations. Suppose in an update, PIR deletes its record. Simply deleting the sequence document would make the data inconsistent with regard to SwissProt Therefore, a series of steps must be enacted to create and delete the appropriate graph elements so that it remains consistent.

However, there are many types of relations in Biozon, and the diverse decisions associated with these operations preclude a single global strategy. To address this problem we define a set of decision making "authorities" as described in the next section. Within this framework, we define protocols for graph update operations of add, delete and modification. This section focuses on the direct response of Biozon to updates. In section 2.5 we discuss the effect of updates on derived data.

Relation authorities

A **relation authority** is any decision-making entity that guards the existence of a relation (edge) in the data graph. An authority is associated with a relation e if it has the ability to prevent deletion of e. Every relation e in Σ must have exactly one authority A associated with it, and every authority A must have at least one edge e associated with it. We denote the authority associated with a relation e as A_e. The authority A_e is only concerned with the documents v_1 and v_2 that are connected by e, and when invoked with an argument v_i it returns 'accept' or 'reject'. If $A_e(v_i) = accept$ then v_i can be deleted as far as e is concerned, without violating internal consistency. Otherwise the deletion is denied.

Each authority is designed to protect consistency in its own limited scope, and has no knowledge of any graph element other than e, v_1 and v_2. Moreover, no authority is aware of the state of other authorities. By dividing a large problem into a number of small solutions and a governing protocol, the problem of consistency of the entire system is tractable, as is described in the next subsection.

As an example, we consider again our representation of SwissProt an instance of which is shown in the main text. In order to maintain internal consistency, we create a relation authority on all instances of the 'describes' relation that are produced from SwissProt Each such relation relates a descriptor document to an amino acid object, and the authority associated with that relation has two rules that govern its response to a request for deletion, namely "deny if the deletion request is on the amino acid and the SwissProt descriptor still exists" and "approve if the deletion request is on the descriptor". This way, the object's existence is always protected as long as the descriptor exists.

Graph modification

Now that we have defined authorities, we present protocols for addition, deletion, and modification of graph elements that preserve internal and external consistency.
Deletion: In our non-redundant graph model where objects are shared by multiple sources, a deletion of a shared object would violate external consistency, and solutions such as foreign key constraints are not applicable in this graph model. The procedure that we define for deletion is an agreement protocol that either allows or disallows the deletion of a given graph part. We define two deletion protocols: one for graph nodes and the other for graph edges.

$delete(v, \mathbf{V})$

Denote by $\mathbf{E}_v$ the set of relations that are associated with the document v, i.e. $\mathbf{E}_v = \{e \in \mathbf{E} | e = (v, u) \texttt{ or } e = (u, v)\}$.

1. For every relation $e \in \mathbf{E}_v$ invoke $A_e(v)$ seeking approval for the deletion of v.

2. If $A_e(v) = reject$ for some $e \in \mathbf{E}_v$ then set $mark(v) = 1$ to indicate that a deletion attempt was made on v, but leave v alive.

3. Else, delete v such that $\mathbf{V} = \mathbf{V} \setminus v$ and invoke $delete(e, \mathbf{E})$ for every relation $e \in \mathbf{E}_v$

$delete(e, \mathbf{E})$

Let v_1 and v_2 the two nodes connected by $e = (v_1, v_2)$

1. Remove relation e such that $\mathbf{E} = \mathbf{E} \setminus e$.

2. If $mark(v_1) = 1$ invoke $delete(v_1, \mathbf{V})$.

3. If $mark(v_2) = 1$ invoke $delete(v_2, \mathbf{V})$.

Note that the two procedures are coupled and dependent on each other. It is also important to note that $mark()$ is an operation that manipulates the value of the 'marked' attribute that is present in every Biozon node (one of the basic three attributes 'docID', 'timeline' and 'marked' that are inherited by *every* document type in Biozon). Therefore, its value may pertain to more than just the current delete operation. Marked nodes, whose attempted deletion was rejected, might be deleted later on once the relation(s) that is associated with a vetoing authority is deleted. Thus, the protocol has built-in garbage collection such that all nodes that have ever had a $delete()$ operation against them will eventually be deleted when it is safe to do so.

Addition: The two issues that need to be addressed when adding an object are non-redundancy and maintenance of derived data. Non-redundancy is handled by the high-level protocol $Add(\sigma, \Sigma)$ described above, and the latter issue is addressed in the next section. Therefore, the low-level $add(v, \mathbf{V})$ and $add(e, \mathbf{E})$ simply add the corresponding element (node or edge) to Σ.

Modification: Modifications to graph elements in Biozon do not exist *per se*. Because of our versioning model with time contexts (see section 2.7) and the shared non-redundant object model, there can not exist an operation that modifies the value of an attribute of a graph element in place. Therefore, the update operation of $v' \rightarrow v$ is modeled as a delete on v' and an add on v. Similarly, a redirection of edges is implemented as a deletion followed by an addition. As such, the act of modifying a document or relation in the database carries with it the problems associated with both addition and deletion. Suppose that we have two databases D and D' and two elements $d \in D$ and $d' \in D'$ such that $T_D(d) \cap T_{D'}(d') \neq \emptyset$, i.e. there exists a document v_i that is common to both subgraphs. Suppose update of D' requires that $v_i \rightarrow u_i$. Simply replacing v_i with u_i in Σ would violate external consistency with D in that $e \in D$ and $d \in \sigma(e)$ but $d \notin \Sigma$ therefore $\sigma(e) \not\subset \Sigma$. To prevent this inconsistency from happening we define $modify()$ in terms of the low-level $add()$ and $delete()$ protocols defined above.

$modify(v', v, \mathbf{V})$

1. Invoke $delete(v', \mathbf{V})$

2. Invoke $add(v, \mathbf{V})$

$modify(e', e, \mathbf{E})$

1. Invoke $delete(e', \mathbf{E})$

2. Invoke $add(e, \mathbf{E})$

2.5 Effect of updates on derived data

Biozon's goals include managing derived data and computations. Therefore, each one of the update operations should be evaluated in the context of this goal. For example, adding new data to the graph needs to be offered as input to the relevant processes. While standard techniques for maintaining materialized views could be employed in most cases, the task may be arbitrarily complex in its execution. For example, computing alignments of newly added proteins and creating subsequent similarity relations between proteins involves comparing the added sequence with the entire library of existing sequences and applying the proper alignment algorithm. Practical limitations dictate that this operation be executed en masse on a cluster of machines. Therefore, there must exist some mechanism to manage the steps required for calculation and instantiating of results.

Deletions might also have consequences on derived data. A particular deletion may affect the input or output of a particular computation. As per the definition of internal consistency, any results that were derived from fully or in part from the deleted data must be removed from the graph if the deletion affects the derived results. We have no *a priori* way of knowing that, however, and only knowledge of the nature of the derivation will give the proper set of actions on the graph to maintain consistency. Similar problems are encountered during modification.

To address these problems we define a set of logical processes we call *graph triggers.* In theory, *all* derived data is generated by graph triggers that monitor the input to the processes that generate the data and are responsible for maintaining consistency with respect to derived data.

Graph triggers

A **graph trigger** is a process that may create or delete elements on the graph. Each graph trigger T operates on a subgraph of Biozon, and is defined over a set $\mathbf{S}(T) \neq \emptyset$ of document classes and relation classes, i.e. $\mathbf{S}(T) = \{c_1, c_2, ..., c_k, c_{k+1}, .., c_l\}$ s.t. $l \geq 1$, where $c_1, c_2, ..., c_k$ are document classes and $c_{k+1}, .., c_l$ are relation classes. The input space of T is composed of all graph elements x such that $class(x) \in \mathbf{S}(T)$. A trigger T is *invoked* whenever there is an addition, deletion, or update on some element in its input space. The trigger's *output* is a set of add() and/or delete() operations on graph primitives. That is to say

$$T(x, o) = \{f_1(v_1), f_2(v_2), ..., f_k(e_k)\}$$

where x is a graph element (node or an edge) in the input space of T and o is the operation that invoked the trigger, i.e. $o \in \{DELETE, INSERT, UPDATE\}$. The output of the trigger it the set $\{f_1(v_1), f_2(v_2), ..., f_k(e_k)\}$ of nodes and edges to add or remove, where $f_i \in \{delete(), add(), modify()\}$.

Deletion

Upon deletion of one of the elements in its input space, the trigger T will evaluate the consequences of that deletion to see if any of the output elements have to be deleted. It should be noted that each trigger T has an authority A_T that is associated with its derived relations. If a $delete()$ operation is called upon one of the nodes connected by these relations, the authority A_T will use the same set of rules that define the trigger T and are used to enforce consistency of derived data. However, authorities that are associated with derived relations are weaker than other authorities and in almost all case will not object deletion attempts.

Addition

If a document is successfully added to the graph, the next step is to evaluate any consequences its addition may have on derived data. The addition of a node v will invoke every trigger T for which v is an instance of a class in $\mathbf{S}(T)$. The subsequent invoked triggers must then perform whatever graph modification operations are implied by the addition of v according to their own definitions.

It should be noted that all operations on D_Σ are considered atomic and occur in one single transaction so that Σ is viewed in a consistent state relative to its constituent databases. With regards to derived relations, however, we can afford to be slightly more loose. Recall the definition of internal consistency given in 2.1. Derived data is inconsistent if there exist a set of derived relations that cannot be derived from data in Σ. For derived data then, the only requirement is that if a graph's modification implies that a certain derivation is now invalid, those graph elements must be deleted. Therefore, we insist that triggers react to delete operations within the same transaction. However, upon addition, there is no explicit requirement that the relevant triggers have to act right away, unless adding a graph node implies deleting existing derived data. Hence, triggers may defer in creating relations in response to graph additions. This is only necessary in some cases where the extreme cost of calculating the data outweighs the negatives of having a temporarily less rich data set. In practice, Biozon exploits this for complicated or expensive operations that are best performed as a batch when there are sufficient computational resources available, such as calculation of protein alignments[6].

Modification

There is one fundamental difference between modification and other operations. The consequences of breaking a modification operation into a separate *add*() and *delete*() is that some unnecessary operations might be activated. Consider the following scenario on an update $v' \to v$ that invokes trigger T. Suppose that the update operation has no effect on the derived relations of T, but the delete and add do. A delete on v' invokes T to delete some graph elements, while an add on v invokes T to add them back. Some effort could be saved if T knew that this was a single update operation. Therefore, the update operation invokes graph triggers *before* it is transformed into separate add and delete operations on the graph primitives.

Examples

The EC mapper. Biozon currently maps proteins to Enzyme families based on the annotations in the descriptors that describe them[7]. Formally, there is a trigger T_{EC} where $\mathbf{S}(T_{EC}) = \{$SwissProt descriptors, PIR descriptors, PDB descriptors, GenPept descriptors, BIND descriptors$\}$. If x is a new descriptor in the input space of T_{EC} that describes protein p then

$$T_{EC}(x, INSERT) = \begin{cases} add(e(EC, p)) & \text{if } x \Rightarrow p \in EC \\ - & \text{otherwise} \end{cases}$$

where $x \Rightarrow p \in EC$ indicates that the descriptor x implies that protein p is in enzyme family EC, and $e(EC, p)$ is a relation ('contains') between enzyme family EC and protein p.

Assume a protein sequence p is described by a descriptor x (say a PIR descriptor) and possibly other descriptors $y_1, y_2, ...$ (SwissProt genpept, etc) with x implying that the protein is a member a specific enzyme family EC. Based on this annotation a relation is formed between the protein and the enzyme family. Assume that at some later time the document x is deleted (but not the other documents). The deletion of this descriptor could cause a trigger to delete the derived relation between the protein and the enzyme family, if there is no other support for this classification. I.e.

$$T_{EC}(x, DELETE) = \begin{cases} delete(e(EC, p)) & \text{if } \neg\exists y \text{ s.t. } class(y) \in \mathbf{S}(T_{EC}) \text{ and } y \Rightarrow p \in EC \\ - & \text{otherwise} \end{cases}$$

[6]Technically, since alignments by BLAST take into account the size of the protein library and use statistics therein to calculate the expectation value, adding a protein or set of proteins may affect every alignment value for existing alignments. However, this change is negligible and is ignored at the moment.

[7]We have also studied other methods to address this problem [Syed and Yona, 2003], however to avoid false relations as much as possible we currently use only human annotations to derive these mappings.

Assume there is an update to $x \rightarrow x'$ then

$$T_{EC}(x, UPDATE) = \begin{cases} delete(e(EC, p)) & \text{if } \neg\exists y \text{ s.t. } class(y) \in \mathbf{S}(T_{EC}) \text{ and } y \Rightarrow p \in EC \\ add(e(EC', p)) & \text{if } x' \Rightarrow p \in EC' \\ - & \text{otherwise} \end{cases}$$

Predicted interactions. As another illustrative example, consider predicted protein-protein interactions. The predicted interactions are generated by analyzing experimental interaction data and similarity data. A predicted interaction between two proteins P_3 and P_4 implies the addition of an interaction object I' and relations between I' and the two interacting proteins P_3 and P_4. Each predicted relation and interaction object depends on both an existing interaction object I and its relations to its constituent proteins P_1 and P_2, and on similarity relationships between proteins, as is depicted in Figure 2. Changes to any of the relevant graph elements must be evaluated to determine if the derived subgraph is still valid. The trigger, then, is invoked whenever there is an operation on interactions, interaction relations, proteins, and similarity relations. There are $4 \times 3 = 12$ possible events (4 classes of graph elements and 3 possible primitive operations), and for the sake of brevity we skip the exact formal description. The trigger output consists of commands that materialize the derived data onto the graph.

As discussed, the derived relation must be associated with the Trigger's authority in order to complete the framework that enforces internal consistency. In this case, the authority would be very simple: they only produce *accept* output, allowing deletion of either the predicted interaction or interacting protein. If the interacting protein is indeed deleted, the authority will allow deletion, and the trigger will destroy the rest of the predicted interaction.

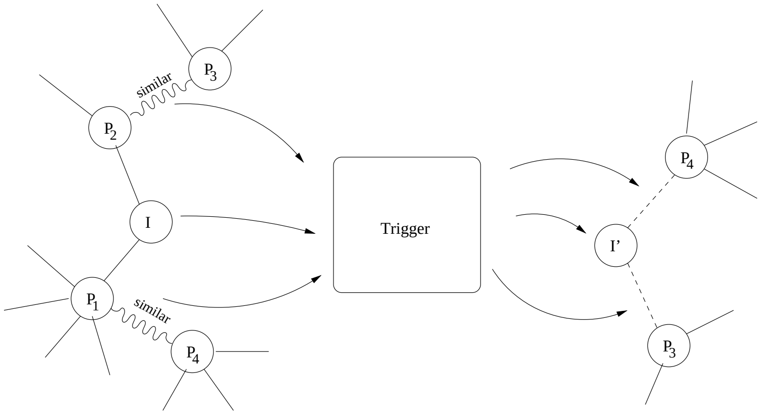

Figure 2: **A graph trigger.**

2.6 Consequences of updates with asynchronous sources

The enforcement of internal consistency (section 2.1) has some interesting effects on the graph structure as events in the constituent databases unfold, particularly in the case of a $Modify()$ operation. One of the biggest issues that can arrive from modifying a database object is a temporary loss of connectivity in the graph. This phenomenon is observed when an update attempt is made on an object that is an element of multiple external databases.

Figure 3 illustrates this situation of connectivity loss. Here we have two objects (Protein and Enzyme family) and three descriptors 1, 2, and 3. The solid lines represent relations that exist between the documents,

and the dotted line represents the provenance of a derived relation (i.e. the derived relation between the protein and EC family was due to annotation in descriptor 3). In the first frame of the illustration, the documents form a cluster around the central protein. In other words, $Desc1$, $Desc2$, and $Desc3$ all come from different source databases, and all three descriptors describe the same protein object.

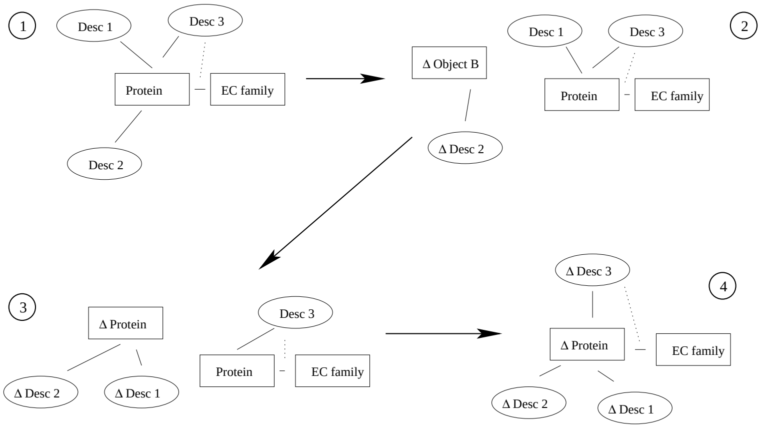

Figure 3: **Splitting of a document cluster due to propagation delay of update**.

In frame 2 of figure 3, we see that see that descriptor 2 and the protein have changed. This could represent a situation where $Desc2$ and the protein are instantiated from a single same element d from database D with $\sigma(d) = (\{Desc2, Protein\}, \{(Desc2, Protein)\})$. Suppose an update to D dictates that $Desc2 \rightarrow \Delta Desc2$ and Protein → ΔProtein. We use the notation ΔProtein and ΔDesc2 to represent the new, updated versions of each of these documents[8].

Now suppose that $Desc1$ is a descriptor from a database, say PDB, and $Desc3$ is yet another, say from Genbank. Let's also suppose none of the databases release their updates simultaneously. Frame 2 of figure 3 reflects the graph after only one update. As the procedures enforcing internal-internal consistency would dictate, a new object $\Delta Protein$ has been added to the database representing the updated object, yet the old protein still exists to satisfy integrity constraints, as it is still live according to the other databases. In frame 3, we have an update of $Desc1$. PDB has just released their update, and they too have discovered the same error as SwissProt and have made the same correction. $Desc3$ still points to the "old" protein, but at least things are still consistent.

In frame 4, $Desc3$ has been updated to reflect the change to the protein published in an update of its original source. Here, we see the cluster of documents has re-formed back into one after a brief stint as two clusters. In reality, though, this convergence might never happen. For instance, two databases might diverge in their definition of an object that they once held in common. This effect is exacerbated in situations where the object is determined by an error-prone process; Furthermore, there might be situations where two descriptors functionally describe the same thing, but the actual makeup of the objects they describe differ slightly or in a trivial way. The technically correct and consistent representation in the database is as two distinct objects that unfortunately serve to mitigate strong connectivity. This problem is remedied somewhat by creating a relation between both versions of the object indicating that they were indeed once the same object. This relation would indicate to the database user that possibly relevant information can be gathered by following the *'was once the same thing'* relation, but indeed places the burden of choice on the user whether or not to follow that relation. Currently, there is no clear general strategy one can take to

[8] For example, suppose Descriptor 2 and its protein originated from a SwissProt document and the sequence is discovered to be in error, so some minor changes are published in the newest release.

resolve these, as each case is unique and requires human intervention.

2.7 Time context

In the discussions of database modifications so far, we have used the term ‘delete’ in the traditional sense, framed in the present time context. To retain the ability to visit the graph at arbitrary times in the past, Biozon adopts an append-only model where data is never really deleted. Instead, we define the time context in which the graph element exists on the graph.

We define a time line as some open or closed interval on the time continuum. Each time line interval begins at a distinct time or infinity and ends at with a distinct time or infinity. A time context will be defined as a point on the time continuum. A database element is considered to exist in a given time context if and only if the point in time representing that time context falls between the start and end point of the time line associated with it. On such a global time scale a delete operation means placing an endpoint on the element’s existence.

Time stamps enable users to access a snapshot of the data as it existed at an arbitrary time context. This is especially important if one would like to reproduce results that were obtained by others sometime in the past. This is a typical problem that users are facing today when using publicly available databases, and often it is impossible to access and verify analysis results that were obtained from subsequently deleted or modified records. Our model addresses this, since it allows users to change the relevant time from present time to an arbitrary point in the past.

2.8 Implementation

For each database D we create a unique transformation function T_D that maps the elements of D onto the Biozon data graph Σ. This function is applied to each element $d \in D$ individually, to generate a subgraph $\sigma(d) \subset \Sigma$. The union of all these subgraphs results in $D_\Sigma = \bigcup_{d \in D} \sigma(d)$

The transformation function T_D is instantiated as a wrapper that parses the data from the source and returns a set of files that together make up D_Σ and are suitable for loading into a relational database. While our current methods are more than adequate for now, we consider using a more unified approach based on formal specification language (in a similar vein to BRIITY) in future versions.

In the data loading and update process, D_Σ is first loaded into a series of temporary pre-loading tables. From there, a stored procedure is executed that actually synchronizes D_Σ with Σ. As such, the responsibilities of this loader are the high-level update procedures including identifying modified documents, assigning docIDs to new elements of D_Σ, expressing relations in D_Σ in terms concrete docIDs, and executing the required SQL commands that insert or update each graph element (which are then handled by low-level update protocols). We create a set of support functions for each data type that perform an equality check for the purposes of eliminating redundancy as well as identifying entries that are modifications to existing entries. These are modular, and are dynamically loaded as needed. This loader, then, implements the high level addition and modification protocols discussed in section 2.3.

We implement the low-level modify and delete operations as described in section 2.4 via triggers which fire on the SQL INSERT, UPDATE, and DELETE commands. Each trigger is fired after the relevant operation on one of the members of its input space (relations or documents). It contains some function that analyzes the operation and the operands and executes the required SQL commands in order to execute that action. Graph triggers may interact with elements outside the DMBS, such as launch external processes or write data files used for computations elsewhere, the results of which are introduced into the database at some later point.

As an illustrative example, consider our implementation of the delete protocol. The low-level delete protocol is handled by a SQL trigger that is fired `BEFORE DELETE`. This trigger queries all the required authorities that are associated with the relations that are connected to the deleted node. If the deletion is

OK, then the SQL trigger allows the deletion process to proceed and changes the timeline timestamp of the node[9]. All graph triggers (section 2.5) are implemented as SQL trigger placed on the delete operation which is performed `AFTER DELETE` (i.e. the node was successfully removed). To implement our protocol of relation deletion in section 2.4 we associate a SQL trigger with each relation. The trigger is fired once the relation is deleted and invokes a `DELETE` operation on each of the two nodes that are connected by that relation, if the node was previously marked for deletion.

2.9 Analysis of a test case

While a comprehensive analysis of the effects and effectiveness of the Biozon update process is beyond the scope of this document, let us consider the update of SwissProt as an example. As of October 2005, the latest update to SwissProt in Biozon occurred on 9 May, 2005 using SwissProt 46.6 that was released on 26 April. A simple analysis of their flatfile reveals a total of 180652 active entries that contain 170734 distinct amino acid sequences. Prior to uploading, Biozon contained an old version of SwissProt that had been through two updates corresponding to previous releases. At the end of the SwissProt update process the number of SwissProt entries in Biozon was found to match the numbers generated from the flatfiles exactly, namely 180652 active descriptor documents and 170734 distinct amino acid described by those documents.

Furthermore, the Biozon graph was searched for protein sequences that were once in the SwissProt database but not in release 46.6. Of those, 4795 were still active on the Biozon data graph. Those 4795 active entries were protected from deletion by the Biozon deletion protocols, implying that each is related to at least one active node that originated from another source. Searching for related database descriptor documents finds that 987 of these protein sequences are currently in the RefSeq database, 83 are in TrEMBL, 96 in BIND, 3276 in GenPept, and 1884 are in PIR. These number might decline upon updates of the individual sources.

[9]This is implemented as a two-step C procedure *below* the SQL delete command, where a 'new' document that represents the 'deleted' form of the document in the proper time context is created first and the old document is then deleted the standard way. This is done to enable after-delete SQL triggers to successfully fire and have the desired effect, in compliance with our consistency protocols.

3 Related studies

Integration of biological databases has been an ongoing research problem that has seen several practical approaches in recent years. This section examines the issues associated with integration and surveys currently existing technologies, highlighting which of our stated goals have been addressed by current research and providing a context for the scope and aims of the Biozon effort.

Prior research by Davidson [Davidson et al., 1995], Spacccapietra [Spaccapietra et al., 1992] and others has identified many of the problems inherent in integrating data from heterogeneous sources into a common schema, and several approaches have been proposed. [Spaccapietra et al., 1992] describe data integration in terms of a two step process of (1) schema investigation and (2) subsequent integration. The investigation step involves examining the input schemas and defining inter-schema correspondences. It does not address this step except to mention that this is typically a manual task. Integration is the task of building a unified schema and then subsequently mapping individual schemas onto it. Davidson goes a step further and describes integration in terms of five necessary steps: (1) Data model transformation, (2) Semantic schema matching, (3) Schema integration, (4) Data transformation and (5) Semantic data matching.

To the best of our knowledge, there are no fully automated methods for arbitrary data model transformation and semantic schema matching. Different methods differ in the extent integration performed, in dealing with different types of conflicts encountered when implementing data integration. They also differ in the methods used to implement integration and the degrees of freedom in the design, including the degree of federation and the choice of warehoused, instantiated data vs views on distributed, independent sources. A detailed discussion of this topic is beyond the scope of this paper and the interested reader is referred to [Davidson et al., 1995].

Current methods for integrating life science data differ greatly in their aims and scope. As was noted in [Hernandez and Kambhampati, 2004], solutions in this area can be given a high level classification into three main categories: portal, mediator, and warehouse.

- "Portal" oriented systems are mainly navigational. These systems perform fast, indexed keyword searches over a flat set but do not actually integrate the data itself. Relationships between data items in these tend to be link driven. Examples of such systems are SRS [Etzold and Argos, 1993] and Entrez [Schuler et al., 1996].
- "Mediator" oriented systems use a mediated schema and/or wrappers to distribute queries amongst different sources, integrating the information in a mediated middle layer. Examples are K2 [Davidson et al., 2001], Kleisli [Chen et al., 2003], TAMBIS [Baker et al., 1998], DiscoveryLink [Haas et al., 2001], BACIIS [Ben-Miled et al., 2003], KIND[Gupta et al., 2000], BioMediator [Mork et al., 2001], BioMoby [Wilkinson and Links, 2002], and Garlic[Haas et al., 2000]. These systems provide a qualified mediated schema onto which sources are mapped, or a single interface or language for access, such as OPM, CPL, or sSQL.
- "Warehouse" oriented systems integrate data into a locally warehoused environment. This is the category that Biozon belongs to, and includes efforts such as GUS [Davidson et al., 2001] and its derivatives, such as AllGenes, PlasmoDB, and EPConDB.

The choice of appropriate technology is determined by the goals that are set forth in its design. Since Portal approaches do not address the problem of data integration we direct the rest of the discussion on the Mediated and Warehouse categories.

3.1 Mediated solutions

3.1.1 Mediated layer

Projects focusing on an integrating mediator layer between the user and heterogeneous data sources have resulted in useful systems. For example, Kleisli [Chen et al., 2003] and DiscoveryLink [Haas et al., 2001] are efforts that attack the integration problem by providing a consistent interface or language that is able to express operations on data from heterogeneous sources. Both provide a language to the user, sSQL (formerly CPL) in the case of Kleisli and standard SQL in the case of DiscoveryLink.

As is typical of most mediated solutions, both Kleisli and DiscoveryLink have source-specific wrappers that provide any necessary access or transformation protocols necessary for creating the interface between the mediating layer and the individual source. Perhaps the most apparent difference between the two is their choice of data model. DiscoveryLink presents a pure relational model to the user, and as such different sources are viewed as a number of relations, each with its own specific set of attributes. Standard SQL operations such as selects or joins may be performed on the data in the various sources. Kleisli, on the other hand, provides a model that goes beyond the flat relational model in that it allows for bulk types that may comprise of sets, bags, and lists [Chen et al., 2003]. Both systems require a detailed knowledge of the structure of the constituent sources in order to use, and neither in and of itself integrates the data into a single, unified schema. Indeed, DiscoveryLink with its emphasis on an SQL interface is oriented more toward the programmer than the biologist [Haas et al., 2001], and the same can perhaps be said of Kleisli, though its extended relational model that uses bulk types may possibly make its use more accessible.

As both DiscoveryLink and Kleisli provide rather 'low level' access to the constituent data, the goals of complex query and extensibility seem to be met rather well by either solution[10]. In the strictest sense of the word, goals of integrating locally-derived results and materializing data may be achieved by creating the appropriate wrappers to include access to locally warehoused data. Indeed, DiscoveryLink and Kleisli are only middle layers and are agnostic of the actual nature of the data they access. Neither solution, however, would inherently address maintaining the warehoused and derived data. Kleisli does provide some features that make it possible to materialize data into an available data store such as an Oracle database [Chen et al., 2003], as well as limited abilities for updating that data. However, the sophisticated manipulations required for maintaining derived data do not exist in its current implementation.

TAMBIS is another mediator-based solution, but differs significantly from the DiscoveryLink and Kleisli efforts in that it provides a high-level interface oriented toward biologists and presents the user with a more unified view of the constituent data. In particular, TAMBIS maps its sources to a global ontology that is used for formulating queries, and as such meets the goal of allowing access to a unified view of the data without specific knowledge on the individual source schemas. This global ontology, however, is not a unified schema. It is mapping of the ontologies in TAMBIS to the underlying schemas of its sources. This ontology-based interface does, however, lend itself rather well to formulating complex queries that can span different domains of data that fit into the underlying ontology.

3.1.2 Mediated schema

Another type of mediated solution is the one implemented in systems such as BioMediator [Mork et al., 2001], or Object Protocol Model [Topaloglou et al., 1999]. These systems use mediated schema on to which independent external databases are mapped.

[10]Complex queries in Biozon adopt a specific meaning as queries that span the relationships between different data types. In that sense, our adoption of a non-redundant object centric model will greatly affect how complex searches are achieved, and the quality and scope of the subsequent results. In that context, neither DiscoveryLink nor Kleisli are completely capable of the types of complex searches present in Biozon. Likewise, since Biozon is not proposing a general purpose query language, there exist queries in DiscoveryLink and Kleisli that are not readily executed in Biozon without knowledge of SQL and Biozon's own internal schema representation.

BioMediator [Mork et al., 2001] addresses the problems of data integration and search by employing a minimal mediated schema. This mediated schema is represented as a graph or network whereby edges between data elements are instantiated as explicit cross-references by accession number, symbol, etc. From this resulting graph, it is possible to determine what sorts of cross-data type queries are possible to execute and the sorts of joins that may be done on these cross-references. All actual data resides in independent published databases themselves, so executing a query invokes a process that formulates a set of queries to send off to each online database and later re-assemble the results into a single result set. The actual degree of integration achieved in Biomediator is limited as semantic schema matching is addressed only to the extent required to do joins across the relevant source datasets. Therefore, their unified schema is intentionally minimal as it is only used in determining plans for query execution. It serves this purpose rather well, and is by design immune from the conflicts that occur with full integration.

Markowitz [Topaloglou et al., 1999] takes a different approach to integration by adopting the Object Protocol Model for representing the structure of the sources. Each source has its own representation in the OPM model, and queries are made with respect to this model in the OPM-MQL language. Meta-data about these models and the correspondences between them are stored in a central mediator, although there is no global mediated schema per se. Data transformation is provided by a set of OPM database servers for each source.

Formal approaches toward mediated schema design were proposed for example by Härder et al. [Härder et al., 1999] who present BRIITY, a view definition language designed to map heterogeneous schema onto a common unified model such as EXPRESS using rules specified in the language. However, nearly all examples of existing data integration solutions do not employ any formal view definition language such as BRIITY as part of their implementation of integration.

3.1.3 Drawbacks of mediated solutions

While mediated solutions are the most common type as of today, they have certain disadvantages. First, there is no guarantee of consistency between independent sources and their references to one another. Efforts using resources such as BioMoby[Wilkinson and Links, 2002], LSID[Object Management Group, 2004] OPM [Chen and Markowitz, 1995], and RDF[Swick and W3C, 1998] to standardize the identification and representation of data have made great strides in making distributed query solutions possible, but have not solved the problems of consistency or performance and have not yet been adopted as a common infrastructure. Indeed, as long as there exist multiple sources for data, there will always be knowledge inconsistencies where two sources may not agree on one fact. Mediated solutions are particularly vulnerable to "dead links" or incorrect links due to versioning inconsistencies. This is analogous to internal consistency as described in section 2.1. Unfortunately, there are no obvious solutions to resolve these in a mediated model.

Furthermore, methods that rely on integrating data from the results of queries to independent data sources are bound by obvious performance constraints. Specifically, with all mediated solutions, efficiency, speed, and availability are all major issues, and are indeed of the largest drawbacks to the mediated model where such performance criteria are significant. Several mediator solutions provide a degree of rule-based or cost-based optimization [Haas et al., 2001]. However, these solutions are still met with practical complications. These inherent scalability issues limit the kinds of operations that can be run within realistic time constraints. Large joins in particular are almost always guaranteed to be slow in non-warehoused environments, and unfortunately these are usually important when executing complex queries over large result sets. The ability to search data using complex queries, which is one of Biozon's most prominent goals, is most practically met in warehouses, where much more control is allowed over optimization.

3.2 Warehouse solutions

Beyond the ability to optimize complex queries, warehousing provides significant benefits that contribute also to the realization of other goals as discussed in this paper. However, there are far fewer warehoused integration systems in existence than there are mediated systems.

Locally instantiated warehouses are essentially materialized views over their sources, and consequently must have in place a system for maintaining these views. Maintenance of views under such conditions has been addressed in works such as [Zhuge et al., 1995] which proposes an appropriate maintenance algorithm and [Engstrom et al., 2003] which discusses maintenance policies. While these methods can be employed to enforce the local warehouse consistency with regard to their external sources, they do not address logical consistency errors between sources where they reference one another. Many databases in the life sciences describe overlapping sets of objects and relationships between objects that may be defined and maintained by other independent entities. These independent sources have little or no coordination in their update schedules or policies. As a result, one observes 'dead links' where one database may refer to another that has been deleted or substantially changed in an update. This issue becomes apparent in semantic data matching, and is especially a problem when integrating data into a model where objects are interrelated and non-redundant. Object identity must be unambiguous, and the existence of any object referred to by a particular source must be guaranteed.

GUS [Davidson et al., 2001] is an example of a warehoused platform upon which projects such as AllGenes are built. Underneath, the data is represented as a normalized relational model. The data in its constituent sources are mapped to a relational schema and stored within GUS. GUS offers a perl-based object layer that is able to provide a layer of abstraction to the underlying relational schemata. While not exactly hiding the details of the schema representation of the underlying databases, it does perhaps make the system more accessible to the user. GUS performs view maintenance techniques in order to synchronize the warehouse contents with its constituent sources, and in addition maintains visioning data on all subsequent changes, and in that sense is quite advanced. However, the goal of maintenance of derived data is not currently addressed.

Moreover, while GUS does provide a single unified global schema comprising of its constituent sources, their translations onto GUS are mostly orthogonal. In other words, sources are incorporated into the GUS schema, but the degree of integration between the sources may be considered loose as defined in [Davidson et al., 1995]. This design choice has implications on searches and navigation and precludes the many benefits of integrated schema as discussed in this paper.

While each approach has its own advantages none of the current efforts in the field are completely effective in achieving all goals we set forth for the Biozon project, as described in the introduction to the main text. To the best of our knowledge, no current integration solution implements integration to its full extent such that the overlapping nature of the data is addressed. Indeed, most existing solutions achieve "horizontal integration", which treats data sources as mostly complimentary, and ignores issues that are associated with data aggregation [Hernandez and Kambhampati, 2004]. One exception is Havasu that considered aspects of vertical integration in their design. Their effort, however, is oriented more toward query optimization in mediated systems rather than aggregating data. Indeed, their research focuses on gathering coverage statistics so as to eliminate queries to irrelevant or less useful sources for a given part of query execution. Biozon, on the other hand, was designed from the start for "vertical integration" which acknowledges the overlapping nature of data, and aggregates the data in one form or another. Adopting a non-redundant object-centric model is useful and has several distinct advantages, as discussed in the main text, and it is one major area that distinguishes Biozon from other current efforts.

References

[Baker et al., 1998] Baker, P. G., Brass, A., Bechhofer, S., Goble, C. A., Paton, N. W., and Stevens, R. (1998). TAMBIS: Transparent access to multiple bioinformatics information sources. In *Proceedings of the Sixth International Conference on Intelligent Systems for Molecular Biology*, pages 25–34.

[Ben-Miled et al., 2003] Ben-Miled, Z., Li, N., Baumgartner, M., and Liu, Y. (2003). A decentralized approach to the integration of life science web databases. *Informatica (Slovenia)*, 27(1):3–14.

[Chen and Markowitz, 1995] Chen, I.-M. A. and Markowitz, V. M. (1995). An overview of the Object-Protocol Model (OPM) and OPM data management tools. *Information Systems*, 20(5):393–418.

[Chen et al., 2003] Chen, J., Chung, S., and Wong, L. (2003). The kleisli query system as a backbone for bioinformatics data integration and analysis. In *Managing Scientific Data*, pages 147–187. Morgan Kaufmann.

[Davidson et al., 1995] Davidson, S., Overton, G. C., and Buneman, P. (1995). Challenges in integrating biological data sources. *Journal of Computational Biology*, 2(4):557–572.

[Davidson et al., 2001] Davidson, S. B., Crabtree, J., Brunk, B. P., Schug, J., Tannen, V., Overton, G. C., and Stoeckert, C. J. (2001). K2/Kleisli and GUS: Experiments in integrated access to genomic data sources. *IBM Systems Journal*, 40(2):512–530.

[Engstrom et al., 2003] Engstrom, H., Chakravarthy, S., and Lings, B. (2003). Maintenance policy selection in heterogeneous Data Warehouse environments: A heuristics-based approach. In *Proceedings of the 6th ACM international workshop on Data warehousing and OLAP*, pages 71–78.

[Etzold and Argos, 1993] Etzold, T. and Argos, P. (1993). SRS - an indexing and retrieval tool for flat file data libraries. *Computer Applications in the Biosciences*, 9(1):59–64.

[Gupta et al., 2000] Gupta, A., Ludascher, B., and Martone, M. E. (2000). Knowledge-based integration of neuroscience data sources. In *Intl. Conference on Statistical and Scientific Database Management*, pages 39–52.

[Haas et al., 2001] Haas, L., Schwarz, P., Kodali, P., Kotlar, E., Rice, J., and Swope, W. (2001). DiscoveryLink: a system for integrated access to life sciences data sources. *IBM Systems Journal*, 40(2):489–511.

[Haas et al., 2000] Haas, L. M., Kodali, P., Rice, J. E., Schwarz, P. M., and Swope, W. C. (2000). Integrating life sciences data-with a little garlic. In *BIBE '00: Proceedings of the 1st IEEE International Symposium on Bioinformatics and Biomedical Engineering*, pages 5–12. IEEE Computer Society.

[Härder et al., 1999] Härder, T., Sauter, G., and Thomas, J. (1999). The intrinsic problems of structural heterogeneity and an approach to their solution. *VLDB Journal: Very Large Data Bases*, 8(1):25–43.

[Hernandez and Kambhampati, 2004] Hernandez, T. and Kambhampati, S. (2004). Integration of biological sources: current systems and challenges ahead. *SIGMOD Rec.*, 33(3):51–60.

[Mork et al., 2001] Mork, P., Halevy, A., and Tarczy-Hornoch, P. (2001). A model for data integration systems of biomedical data applied to online genetic databases. In *Proceedings of the 2001 AMIA Annual Symposium*, pages 473–477.

[Object Management Group, 2004] Object Management Group (2004). Life science identifiers: OMG final available life science identifier specification. http://www.omg.org/cgi-bin/doc?dtc/04-10-08.

[Schuler et al., 1996] Schuler, G. D., Epstein, J., Ohkawa, H., and Kans, J. A. (1996). Entrez: Molecular biology database and retrieval system. *Methods in Enzymology*, 266:141–161.

[Spaccapietra et al., 1992] Spaccapietra, S., Parent, C., and Dupont, Y. (1992). Model independent assertions for integration of heterogeneous schemas. *VLDB Journal*, 1(1):81–126.

[Swick and W3C, 1998] Swick, R. R. and W3C (1998). Resource description framework (RDF) model and syntax. http://www.w3.org/TR/WD-rdf-syntax.

[Syed and Yona, 2003] Syed, U. and Yona, G. (2003). Using a mixture of probabilistic decision trees for direct prediction of protein function. In *Proceedings of the seventh annual international conference on Computational molecular biology (RECOMB-03)*, pages 289–300.

[Topaloglou et al., 1999] Topaloglou, T., Kosky, A., and Markowitz, V. (1999). Seamless integration of biological applications within a database framework. In Lengauer, T., Schneider, R., Bork, P., Brutlag, D., Glasgow, J., Mewes, H.-W., and Zimmer, R., editors, *Proceedings of the 7th International Conference on Intelligent Systems for Molecular Biology, ISMB'99 (Heidelberg, Germany, August 6-10, 1999)*, pages 272–281, Menlo Park. AAAI Press.

[Wilkinson and Links, 2002] Wilkinson, M. D. and Links, M. (2002). BioMOBY: An open source biological web services proposal. *Briefings in Bioinformatics*, 3(4):331–341.

[Zhuge et al., 1995] Zhuge, Y., García-Molina, H., Hammer, J., and Widom, J. (1995). View maintenance in a warehousing environment. In *ACM SIGMOD '95*, pages 316–327.
